# Conformational Heterogeneity Underlying Divergent Signaling in Class A G Protein-Coupled Receptors

**DOI:** 10.1021/acsptsci.5c00102

**Published:** 2025-10-07

**Authors:** Kyriakos Georgiou, Antonios Kolocouris

**Affiliations:** Laboratory of Medicinal Chemistry, Section of Pharmaceutical Chemistry, Department of Pharmacy, School of Health Sciences, 69232 National and Kapodistrian University of Athens, Panepistimiopolis-Zografou, 15771 Athens, Greece

**Keywords:** A_2A_ adenosine receptor, β_1_ adrenergic receptor, β_2_ adrenergic receptor, μ opioid recerptor, conformational equilibrium, transient conformations

## Abstract

Class A G protein-coupled receptors (GPCRs) are targets for ∼36% of commercial drugs. GPCRs in their apo-forms exhibit conformational heterogeneity, and more than a single active and inactive conformation exists in equilibrium. Distinct transient conformational states can be significantly populated and can be coupled with different agonists, transducers, and effectors, giving rise to divergent signaling pathways. The characterization of such transient conformational states, which may have eluded identification by X-ray crystallography and cryogenic electron microscopy, can be achieved through a combination of biophysical techniques, such as nuclear magnetic resonance, double electron–electron resonance spectroscopy, single-molecule fluorescence microscopy, molecular dynamics simulations, and mass spectrometry. We review findings about the functional, conformational states of four class A GPCRs, including detailed results for the adenosine A_2A_ and β_2_ adrenergic receptors and important observations for the β_1_ and μ opioid receptors. The identification of ligands that can bind to distinct conformations, e.g., agonists that activate favorable pathways while inhibiting deleterious ones, represents an important goal in drug development.

## Graphical abstract

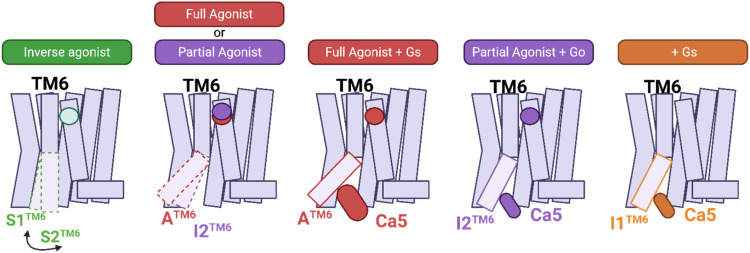

## 1 Purpose of the Review

On the question of which conformations of a G protein-coupled receptor (GPCR) are best for a given transducer protein coupling that can activate one signaling pathway over another, an answer cannot be given in general. The structural hallmarks that promote GPCR coupling to other G proteins and signal transducers, including arrestin (arr) proteins and GPCR kinases (GPCRKs, GRKs), will undoubtedly be revealed by structural biology studies. Research aimed at characterizing the conformational properties of GPCRs is crucial not only for structural and molecular biologists and biophysicists but also for scientists working to translate this knowledge into new drug development.^1–8^ In this review, we focus on results showing the complexity of describing the conformational landscape and signaling of the β_2_ adrenergic receptor (β_2_AR), adenosine A_2A_ receptor (A_2A_R), as well as the β_1_ adrenergic receptor (β_1_AR) and μ opioid receptor (μOR). These receptors have been studied, for example, with X-ray crystallography, cryogenic electron microscopy (cryo-EM), biomolecular simulations, nuclear magnetic resonance (NMR), and electron–electron double resonance (DEER) spectroscopy, single-molecule fluorescence (SMF) microscopy, single-molecule fluorescence resonance energy transfer (smFRET), and mass spectrometry (MS) techniques applied with GPCRs, e.g., hydrogen–deuterium exchange MS (HDX-MS) and hydroxyl radical footprinting MS (HRF-MS), high-throughput matrix-assisted laser desorption/ionization MS (MALDI-MS), or native MS (nMS). Recall that β_2_AR is the target of both antagonists^9^ and partial and full agonists^10^ for the treatment of cardiovascular and respiratory diseases, while A_2A_R antagonists show promise in Alzheimer’s and Parkinson’s diseases, attention-deficit hyperactivity disorder, depression, and anxiety, while A_2A_R agonists could be used in Niemann Pick type C disease, autism-spectrum disorders, and schizophrenia.^11^ Targeting the β_1_AR has several important therapeutic benefits, especially in cardiovascular medicine. This receptor subtype is primarily located in the heart and plays a key role in regulating heart rate and contractility in response to catecholamines like norepinephrine and epinephrine.^12^ Agonists of the μOR, such as opioid analgesics like morphine, can reduce pain by activating these pain receptors in the central nervous system (CNS) that induce G protein-mediated signaling to confer analgesia; however, they can also cause β-arr activation, which can result in adverse consequences, including respiratory depression. Pain and adverse effects can be reduced by biased ligands to μOR that trigger G protein signaling without triggering β-arr.^13^ Even for these characteristic class A GPCRs, the description of conformational heterogeneity, i.e., the elucidation and understanding of the role of distinct conformations in signaling, is a challenging task since the GPCRs also exhibit divergent conformational behavior.^14^

## 2 Background

### 2.1 G Protein-Coupled Receptors

GPCRs constitute over 1% of the human genome and are extremely important for physiological function. Human cells express 826 distinct GPCRs across all organ systems,^15^ making them the biggest family of cell surface proteins. It is worth noting again that “the discovery of the close structural relationship between rhodopsin receptor (RhoR) and β_2_ adrenergic receptor (β_2_AR), and of the existence of a larger “superfamily” of such receptors, came as a total surprise,” as commented by Lefkowitz in 2000.^16^ Class A GPCRs represent targets for approximately 36%^17^ of commercial drugs.^18–20^ Based on conserved sequence and similarity signatures, human GPCRs are categorized into A (rhodopsin-like family), B (secretin family), B2 (adhesion family), C (glutamate receptors), and F (Frizzled receptors) subfamilies. GPCRs are membrane proteins with seven transmembrane α-helix (7TM) domains connected by three extracellular loops (ECL 1–3) and three intracellular loops (ICL 1–3). Thus, GPCRs have a 7TM with most of them featuring an additional intracellular helix 8 (H8) connected at the end of TM7 and a disordered C-terminal tail (C-tail). GPCRs span plasma membranes, with glycerophospholipids (phospholipids)^21^ being the most abundant lipids.

When an agonist binds to a class A GPCR in an extracellular pocket, the orthosteric binding site (OBS), which is the primary binding pocket of an endogenous ligand in the receptor’s core segment TM3-TM4-ECL2-TM5-TM6, produces a conformational transition of amino acid residues that are coupled with the intracellular/cytoplasmic region of the GPCR.^3,7,22–26^ This “microswitch” motif network generates an intracellular cavity, usually through pivotal and outward movement of TM6 as regards the 7TM bundle core, that binds and activates transducer proteins, such as G proteins or arrs^27–31^ or GRKs,^32^ which in turn recruit selective signaling effectors to regulate many physiological processes.^3^

There are 16 human Gα genes,^33^ and the G proteins are classified into four subtypes based on the Gα subunit (grouped into the Gs, Gi/o, Gq/11, and G12/13 subfamilies).^34^ Nonetheless, coupling to Gi/o (these include Gi1, Gi2, Gi3, and Go, and are collectively known as Gi/o), Gs, or Gq is often used for classification as regards coupling to GPCRs. Heterotrimeric G proteins are formed when the latter proteins combine with Gβ and Gγ proteins. When GPCRs activate the G-protein complex, it disassembles, and the separate subunits can then trigger distinct signaling pathways. For example, cyclic AMP molecules, which control several cellular functions, are elevated in cells by stimulatory Gα proteins, or Gs proteins. A GPCR can typically activate several G proteins and the corresponding signaling pathways, and each G protein can interact with several different GPCRs^35^ and the comparison of the GPCR–G structures shows significant structural plasticity at the interface.^36^ Activated GPCRs can be phosphorylated by GRKs and attached to arrs in parallel to G protein signaling, triggering GPCR desensitization, GPCR endocytosis, and arr-dependent signaling.^37–39^ Mammals widely express beyond the 16 G protein subtypes, four arr subtypes (arr2 and arr3), and 7 GRKs (GRK2, GRK3, GRK5, and GRK6).

### 2.2 Signaling Complexes of Class A G Protein-Coupled Receptors

#### 2.2.1 Coupling with G Proteins

##### 2.2.1.1 Activation of GPCRs

During activation, a loosely coupled allosteric network of residues^40–42^ is formed spanning the 7TM bundle, including the OBS, the connector/transmembrane region, and the cytoplasmic region. Each of the three regions (Figure 1) can switch between many distinct conformations, leading to the fully activated conformation of GPCR. The small perturbations at the extracellular OBS drive substantial conformational changes at the cytoplasmic G protein binding site. A study of ∼230 structures of 45 class A GPCRs revealed a set of 34 amino acid residue pairs that contribute to the activation pathway.^43^ As analyzed in several articles by Kobilka and collaborators,^3,22–24^ or Venkatakrishnan and Babu,^44^ or Glukhova, Sexton, and collaborators,^30^ for the activation of a class A GPCR by an agonist, amino acid residues that belong to or correlate to a motif network^45^ should adopt certain conformations. This implicated the canonical “microswitch” motif network^45^ includes the C^6.47^W^6.48^P^6.50^ (CWxP) motif,^46^ the P^5.50^I^3.40^F^6.44^ (PIF) motif,^47^ the Na^+^ pocket,^48^ the D^3.49^R^3.50^Y^3.51^ (DRY) motif,^49^ and the N^7.49^P^7.50^xxY^7.53^ (NPxxY) motif^50^ along with the conserved Y^5.58^ residue in TM5 included by the T^3.46^Y^5.58^Y^7.53^ polar motif (Ballesteros-Weinstein numbering^51^ in superscript). These motifs have coupled conformational motion. The time needed for the activation of the receptor can be several milliseconds (ms), as was shown by Chung, Kobilka, Lodowski, and collaborators.^26^

**Figure 1:**
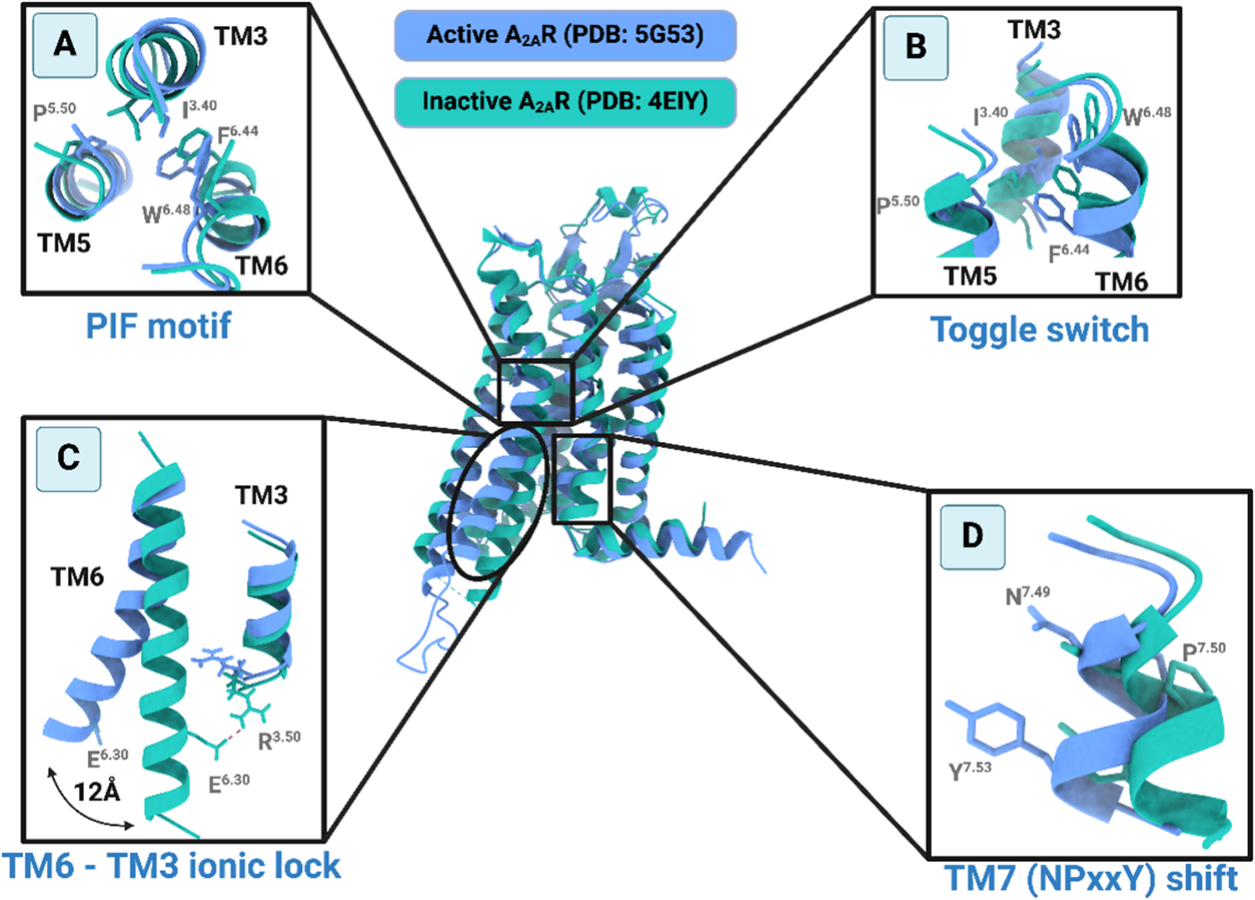
Main conformational changes that must occur during GPCR activation/inactivation shown for A_2A_R by comparison of the conformation of A_2A_R in its fully active state as revealed in the X-ray structure of agonist 5′-*N*-ethylcarboxamidoadenosine (NECA)–A_2A_R–mini-Gs (PDB ID 5G53 ^56^) mini-Gs is an engineered truncated Gs protein and in its inactive state in the complex of the inverse agonist ZM241385 (PDB ID 4EIY^57^). Blue and green cartoons depict the A_2A_R’s active and inactive conformations, respectively. In the insets are shown four main microswitches of class A GPCRs:^3,22–24,44,45^ (A) the “toggle switch” residue W^6.48^ in the OBS/connector TM region, which changes side chain rotamer state (top view); (B) the coupled motion of the residues in PIF motif in the TM connector region (side view); (C) the TM6-TM3 inactivating “ionic lock” in the intracellular region close to the binding surface of G protein (side view) and the outward displacement of the cytoplasmic TM6 end; (D) the NPxxY inward/outward shift in the intracellular region close to the binding surface of G protein (side view). The residues that are significantly implicated in these conformational transitions are shown as sticks in the panels. Hydrogens are omitted for the sake of clarity (figure inspired by ref 58).

In more detail, the agonist binds to the OBS and causes W^6.48^, in the C^6.47^W^6.48^P^6.50^ motif,^46^ to move lower, toward residue F^6.44^ (conserved in 82% of class A GPCRs) of the P^5.50^I^3.40^F^6.44^ motif^47^ (which is highly conserved in class A GPCRs), initiating the rotation of TM6′s cytoplasmic end. In the fully activated receptor, F^6.44^ forms stabilizing interactions; residues P^5.50^ and F^6.44^ in a cis position form a CH-π hydrophobic interaction, while the CH_3_ group of I^3.40^ similarly forms a CH_3_-π interaction. The packing rearrangement in I^3.40^, P^5.50^, L^5.51^, and F^6.44^ motifs weakens TM5/TM6 contacts, and the change at P^5.50^ rotamer induces the subsequent signal transduction, which is transmitted from TM5 to TM6. In particular, in the C^6.47^W^6.48^P^6.50^ motif, P^6.50^ acts as a hinge in the TM6, reducing the activation energy barrier to the opening of the intracellular cavity. Thus, the small rearrangements of the conserved PIF motif are allosterically linked^40–42^ with the outward displacement of TM6.^3,22–24,30,44^ A water-filled cavity surrounding D^2.50^ contributes to the stabilization of a sodium cation as observed in several crystal structures of GPCRs in the inactive state. This sodium-binding site allosterically stabilizes TM3 and TM7 in the inactive state. The motion of TM5 causes the putative sodium-binding pocket to collapse,^48^ leading to dehydration of D^2.50^ and displacement of the sodium cation, which is free to egress in the cytosol^52^ and a possible protonation of D^2.50^.^53^ Thus, the TM5 motion results in contacts between N^7.49^ in the intracellular region, in the N^7.49^P^7.50^xxY^7.53^ motif,^47^ and N^7.45^, D^2.50^, S^3.39^, causing TM7 to migrate toward TM3. The full activation in the ternary complex agonist-GPCR-G causes TM6 to move outward and TM7 to rotate with the inward movement of the NPxxY^7.53^ motif, allowing Y^7.53^ to lose contact with residues in TM1 or H8 and shift toward TM3, enhancing TM3-TM7 packing. Y^7.53^ adopts the unique active state conformation by forming the strong Y^5.58^---W^6.48^---Y^7.53^ or Y^5.58^---water---Y^7.53^ hydrogen bonding interaction (Y–Y interaction^54^), strengthening the TM5-TM7 packing and stabilizing the outward shift of TM6.^3,22–24,3044^ Recent research by Ye, Cheng, Miao, and collaborators in A_2A_R reveals that the stabilizing R^8.48^-H^6.32^ cation-π interaction is observed in the fully activated conformation, while for the stabilization of the inactive conformation, except for the ionic lock DR^3.50^Y-E^6.30^, it is necessary the cation-π R291^7.56^-H230^6.32^ interactions.^55^ In ref 55, it was also shown that mutation R291^7.56^A traps A_2A_R in an intermediate state that prevents the receptors from adopting the fully activated state.

The “ionic lock” DR^3.50^Y-E^6.30^ interaction,^59^ which is observed in the X-ray structures of the inactive state of bovine RhoR^60^ and the inactive state of A_2A_R,^61^ is disrupted in the active conformation of the ternary complex as a consequence of the outward translation of TM6 from TM3. Such movement can be observed, for example, by comparison of the XFEL structure of inactive RhoR, i.e., bovine RhoR with 11-cis retinal, reported by Okada, Buss, and collaborators in 2004 ^60^ (PDB ID 1I19 ^60^) and the cryo-EM of fully active RhoR (bound to inhibitory G protein Gi) reported by Xu, Subramaniam, Kossiakoff, and collaborators in 2018 ^62^ (PDB ID 6CMO^62^). However, this R^3.50^-E^6.30^ “ionic lock” interaction is not present in the X-ray structures of the inactive conformation, e.g., of β_1_AR and β_2_AR.^63–67^ Indeed, the equilibrium of an inactive conformation with R^3.50^-E^6.30^ “ionic lock” with an inactive conformation with broken R^3.50^-E^6.30^ “ionic lock” was observed by solution ^19^F NMR in A_2A_R reconstituted in micelles, as reported by Prosser, Ernst, and collaborators in 2016,^68^ and by solution ^19^F NMR in β_2_AR reconstituted in micelles, as reported by Kobilka and collaborators in 2015 ^69^ and discussed afterward. Thus, the DR^3.50^Y-E^6.30^ interaction does not differentiate the inactive from the active conformational ensemble since it occurs during the transition between two inactive states, which is required for the activation of these class A GPCRs.^55^

Despite the highly conserved allosteric motifs network, GPCRs can be differentiated even in the TM6 outward movement. Thus, Kobilka, Skiniotis, Mathiesen, and collaborators reported in 2020 ^70^ on the different activation mechanisms between GPCR class A β_2_AR and class B glucagon receptor (GCGR). Both agonist and G protein binding are required for the receptor to move toward an active state in both receptors. However, although an outward movement of TM6 due to agonist binding is a key characteristic in the activation of β_2_AR, in GCGR, the TM6 shows an a-helix disruption and a sharp kink formation, resulting in much slower kinetics of activation.^70^

A work that challenges the typical model of receptor antagonism and offers critical insights into GPCR pharmacology was published by Gati and collaborators in 2025.^71^ It was shown^71^ that certain inverse agonists of the κ-opioid receptor (κOR) can function through κOR-Gi protein complexes. Strikingly, three cryogenic electron microscopy (cryo-EM) structures of κOR-G_i_ protein complexes with different inverse agonists (norBNI, JDTic, GB18) showed that the complexes of inverse agonist-GPCR are also bound to Gi proteins (PDB IDs 8VVE, 8VVF, 8VVG^71^). Remarkably, the OBS has an inactive receptor conformation, yet the receptor stays attached to the Gi protein.

The understanding of the structure and function of GPCRs, as well as the development of drugs against GPCRs, has been greatly increased by advances in structural biology.^72–77^ Overall, as regards the static structures, there are now more cryo-EM structures (623 structures) than X-ray structures (477 structures), while most cryo-EM GPCR structures are available, due to the size requirements of the protein, in the fully activated conformation of the GPCR, according to data selected in the GPCRdb (GPCR database).^78,79^ However, most recently, an agonist–GPCR–G protein complex with GPCR (R291^7.56^A A_2A_R) in an intermediate active conformation was resolved by the collaborative effort of the laboratories of Ye, Cheng, Miao, and reported in 2025 (PDB IDs 9EE8, 9EE9, 9EEA^80^).

##### 2.2.1.2 Activation of G Proteins

GPCRs can transmit extracellular signals by activating heterotrimeric Gαβγ proteins consisting of the α-, β-, and γ-subunits (Gα, Gβ, and Gγ, respectively). A graphical description is shown in Figure 2.

**Figure 2:**
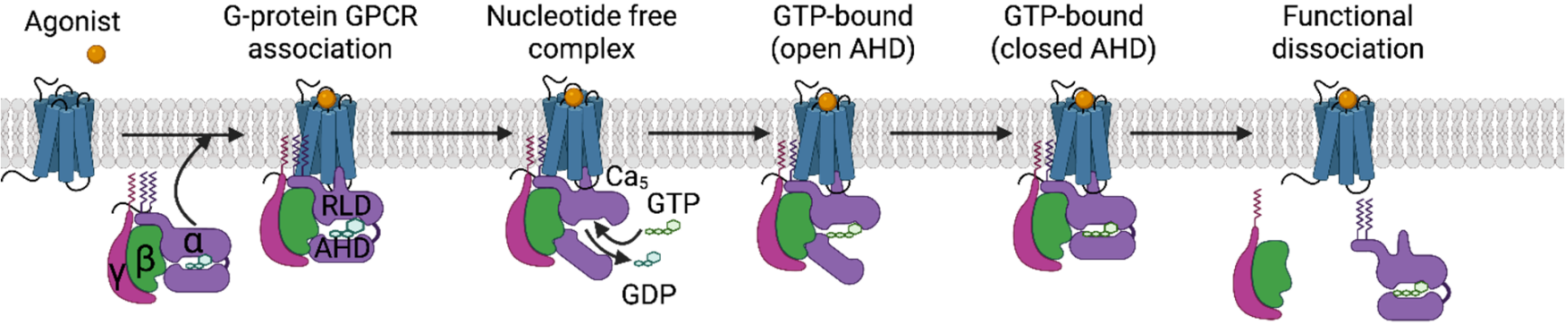
Conformational changes that Gα undergoes to shift from the Gα protein coupling, bound GDP, and GDP dissociation to the empty GDP (guanosine diphosphate, GDP) state and GTP (guanosine triphosphate, GTP) binding, which results in G protein heterotrimer activation, dissociation of Gβγ, and separation from the GPCR.^26,81–87^

The Gα subunit has GTPase activity. The two domains that make up the structure of all Gα subunits are the conserved (over small GTPases) nucleotide-binding Ras-like GTPase domain (also called the GTPase domain or G domain) and the α-helical domain (AHD), forming a lid over the nucleotide-binding pocket. The Ras domain is in contact with Gβγ proteins, the GPCR, and effector proteins. Both mini-G proteins and the Gα of heterotrimeric Gαβγ contain a GTPase domain, while Gα contains in addition the AHD and forms a complex with the Gβ and Gγ subunits. For the nucleotide unbinding, the Ras domain must be separated from its connection with AHD, combined with conformational changes of Ras regions that interact with the nucleotide. Large conformational changes can be observed between the GDP- and GTP-bound structures of the Ras-like domain (RHD).^88^ The Gβ subunit consists of an α-helix at the N-terminus connected with seven WD40 repeats that combine to produce a seven-bladed β-propeller. The Gγ subunit has a single helix structure that is connected to Gβ to create an obligate Gβγ dimer. Both Gα and Gβγ anchor in the membrane. The Gγ is prenylated at the C-terminus, while the Gα at the N-terminus is attached to a palmitoyl or myristoyl group.^89^

When an agonist binds to OBS, it causes an intracellular conformational change^22,23,31,81,90–93^ that allows receptor binding to the GDP-bound Gα (Gα^GDP^) subunit of the heterotrimeric G protein.^94,95^ In the agonist–GPCR–Gα complex, GPCR lies in an active conformation. The outward opening of TM6 observed in the active states of agonist–GPCR–G protein ternary complexes opens the receptor’s cytoplasmic cavity for G protein coupling.^30^ It has been demonstrated that Gα activation follows a highly conserved allosteric mechanism, considering that there are over 800 distinct GPCRs and 16 distinct Gα genes in humans, as analyzed by Flock and Babu in 2015.^86^ This mechanism was explored in complexes of class A GPCRs with Gs protein, e.g., with β_2_AR, using X-ray crystallography as reported by Kobilka, Sunahara, and collaborators in 2011 ^82^ or Skiniotis, Kobilka, Sunahara, and collaborators in 2011;^83^ using HDX-MS reported by Sunahara, Woods, Kobilka, and collaborators in 2011;^84^ with A_2A_R using cryo-EM reported by Tate and collaborators in 2018;^85^ with RhoR using DEER spectroscopy reported by Hubbell, Hamm, Miller, and collaborators in 2006,^88^ Hamm, Hubbel, and collaborators reported in 2007,^96^ or Hubbell, Hamm, Miller, and collaborators reported in 2011.^87^ Then, Blanchard, Kobilka, and collaborators reported in 2017,^97^ smFRET results in β_2_AR; Chung, Kobilka, Lodowski, and collaborators published in 2019,^26^ time-resolved MS results in β_2_AR and A_2A_R; Skiniotis and collaborators published in 2024,^81^ time-resolved cryo-EM results in β_2_AR; Ye, Cheng, Miao, and collaborators reported in 2025 ^80^ using a combination of solution ^19^F NMR, cryo-EM, and MD simulations in A_2A_R, inspired the feasibility of capturing a GPCR-G intermediate during the activation process. It has been observed that the C-terminal helix of Gα (Cα5 helix) in Gα^GDP^ in the Ras-like domain plays a critical role in the GPCR-G protein interaction. The Cα5 helix, and particularly the distal C-end part (known as the “wavy hook”), must be inserted into the receptor’s cytoplasmic cleft to couple with the GPCR,^26,81–87^ whereas the N-terminal helix (helix N) of Gα interacts with H8 of the receptor and Gβγ proteins at the membrane interface. Residue R^3.50^ forms polar interactions with the Cα5 helix of the G protein as part of a polar contacts network of the Cα5, mainly with TM3, TM5, and ICL2 of the cytoplasmic cavity. A clockwise rotation of TM6 and counterclockwise rotation of Cα5 helix complete the formation of the compact, fully activated complex stabilized by the formation of a strong ionic hydrogen bonding interaction R^7.56^-E392­(Gα) and a strong cation-π interaction R^3.50^-Y391­(Gα).^26,81,86^

The Cα5 helix requires a conformational shift to couple with the GPCR.^26,81–87^ Rearrangement of the Cα5 helix (lift toward the cytosolic cavity of GPCR) caused its detachment from the Gα H1 helix in the Ras-like domain and resulted in a decreased affinity of GDP, which dissociated from the G protein to produce the nucleotide-free Gα (Gα^empty^) or inactive G protein. Then, GTP rapidly binds Gα to the Gα^empty^, in a closed conformation between RHD and AHD, causing conformational changes in RHD, which dissociates Gα from the receptor and Gβγ subunits.^26,81–87^ After dissociation, the GTP-bound Gα (Gα^GTP^) and free Gβγ subunits are fully activated and can control downstream signaling effectors, which ultimately lead to certain cellular phenotypes/behaviors. For example, Gα^GTP^ signals through phospholipase Cβ, adenylyl cyclase (AC), and RhoGEFs; RhoGEF domain describes two distinct structural domains with guanine nucleotide exchange factor (GEF) activity to regulate small GTPases in the RhoR family. The Gβγ subunit interacts with phosphatidylinositol-3 kinase, mitogen-activated protein kinases, calcium channels, and voltage-gated potassium channels, but also with phospholipase Cβ and AC.^98^

The signal transduction relies heavily on the allosteric interaction between the ligand, GPCR, G protein, and GDP/GTP. There is a reciprocal cooperation between the orthosteric agonist binding and G protein coupling that is inherent to all GPCRs, as suggested in the early study by De Lean and collaborators in 1980,^99^ and afterward reported, for example, by Hamm and Hubbel in 2007.^96^ When GDP is released from Gα, this cooperation is further enhanced, resulting in a nucleotide-free, high-affinity ternary complex GPCR-G^empty^ with a minutes-lifetime in a free GTP environment. Current techniques for quantifying nucleotide exchange and GTP hydrolysis of individual G proteins depend on calorimetry- and radioactive substrate-based assays, or fluorescence using tagged substrate analogs or protein assays, or NMR-based methods through ^13^C, ^15^N, or ^19^F labeling of G protein (see ref 100 and references therein). The hydrolysis of GTP to GDP, which is carried out either through Gα’s subunit intrinsic GTPase activity or in cooperation with G protein signaling modulators, inactivates Gα, allowing Gα and Gβγ subunits to reassociate to the heterotrimer^101,102^ (Figure 2). Thus, Gαβγ proteins function as molecular switches.^34^ After prolonged GPCR activation, GRKs phosphorylate the receptor, which then couples to β-arr.^103^ Desensitization and arr-mediated activation of downstream effectors are the outcomes of this coupling.^104^ The receptor eventually internalizes into the endosome, where it is degraded or dephosphorylated and recycled back into the plasma membrane.^103^

#### 2.2.2 Coupling with β-arrs

The structures of GPCR-β-arr complexes showed that β-arr primarily interacts with TM7, H8, the phosphorylated ICLs, and possibly the C-terminal tail of a GPCR, compared to Gα that mostly interacts with cytoplasmic portions of TM3, TM5, TM6, and ICL2, ICL3.^105^ These observations were made based on the available experimental structures. Thus, it has been shown that an arr binds to GPCRs with a tail conformation or with a core conformation responsible for internalization or desensitization, respectively.^106^ Studies with several different GPCRs suggested that TM7 mediates signaling bias and receptor coupling to β-arr, such as structural studies by Roth, Corvy, and collaborators with the human serotonin 1B receptor (5-HT_1B_R),^107^ or Roth, Wacker, and collaborators with κOR,^108^ Xu and collaborators with RhoR;^109^ fluorescence studies by Granier, Mouillac, and collaborators with arginine-vasopressin type 2 receptor (V2R);^110^ solution ^19^F NMR by Wüthrich, Stevens, and collaborators with β_2_AR.^111^

Representative examples of β-arr coupling through a tail conformation are those provided by Lefkowitz and collaborators, published in 2017 ^106^ in the study of the β_2_AR-βarr1 complex using negative-stain electron microscopy or the high-resolution cryo-EM structures of the complexes of glucagon receptor (GCGR)-βarr1 without or with glucagon agonist (PDB IDs 8JRU and 8JRV,^112^ respectively) reported by Wu, Zhao, and collaborators in 2023.^112^ Even with the glucagon agonist present, the GCGR-βarr1 complex assumes a conformation of GPCR more like the inactive state than the active one^112^ possibly because the agonist by itself is unable to completely maintain the active conformation in the absence of the transducer to enlarge the intracellular pocket.

In contrast, in other available structures of GPCR-arr complexes, the arr core binds in the GPCR intracellular cavity, which maintains an active state.^105,113–117^ Such examples are the X-ray free electron laser (XFEL) crystal structure of RhoR-arr complex (PDB ID 5W0P^115^) reported by Xu and collaborators in 2017;^115^ the cryo-EM structure of the human muscarinic acetylcholine receptor type 2 (M2R) in complex with agonist iperoxo, PAM LY211960, and βarr1 (PDB ID 6I1N^114^) reported by Skiniotis, Lefkowitz, and collaborators in 2020;^114^ the cryo-EM structure of the human neurotensin receptor 1 (NTS1R) in complex with agonist NTS_8–13_ and βarr1 (PDB ID 6UP7 ^113^) reported by Xu and collaborators in 2017;^113^ the cryo-EM structure of the β_1_AR from turkey in complex with agonist formoterol and βarr1 (6TKO^105^) reported by Tate and collaborators in 2020.^105^ Additionally, the X-ray structures of the human angiotensin II (AngII) receptor type 1 with only bound to the balanced AngII, which is the endogenous agonist, or with each of the two strongly β-arr-biased agonists TRV023, TRV026, reported by Kruse, Lefkowitz, and collaborators in 2020 ^118^ (PDB IDs 6OS0,^118^ 6OS1,^118^ 6OS2,^118^ respectively). The MD simulations of the complex of AT1R with AngII published by Dror and collaborators in 2020 ^119^ led to similar observations.

#### 2.2.3 Coupling with GRKs

On the question of which conformations are best for a given transducer coupling, it is important to note that while a few high-resolution structures of GPCR-arr complexes have been deposited, only two exist for GPCR-GRK complexes. For example, the structure of light-activated RhoR in complex with GRK1 (PDB ID 7MT9 ^120^) was published by Tesmer and collaborators in 2021.^120^

Another example is the structure of the NTS1R–GRK2-Gα complex (8JPB, 8JPC^121^) reported by Duan, Yang, Xu, and collaborators in 2023.^121^ The N-terminal helix (αN helix) of GRK2 binds into the open cytosolic cavity formed by the outward movement of TM6, analogous to the binding of the G protein to the receptor. The binding site of G protein to NTS1R, which is made up of ICL2, TM6, TM7, H8, and the major binding site of GRK2 at NTSR1, shares characteristics that are very comparable in the active structures of other GPCRs.

#### 2.2.4 Coupling with Multiple Transducers

Structures of a class A GPCR in complex with different transducer proteins are very useful for comparison reasons and for exploring biased signaling. Thus, the structure of the inactive NTS1R in complex with antagonist SR48692 and a universal nanobody, such as Nb6 (nanobody/Nb is a single domain camelid antibody fragment), was reported by Skiniotis and collaborators in 2024,^122^ (PDB ID 7UL2 ^122^) the structure of the NTS1R−β-arr2 complex (PDB ID 6UP7 ^113^) was reported by Kobilka, Skiniotis, and collaborators in 2020,^113^ and the structure of agonist peptide JMV449–NTS1R–Gi1 complex (PDB ID 6OS9 ^123^) was reported by Skiniotis, Kobilka, and collaborators in 2019.^123^

The pharmacological analysis showed that SBI-553 is a unique allosteric modulator against NTS1R, possessing a broad spectrum of allosteric effects. Thus, it is a biased negative allosteric modulator (NAM) with activity at Gq > G15 > Gi ≫ G12 and PAM-agonist activity as regards the endogenous neurotensin peptide (NTS) for β-arr recruitment.^124^ In the same context, interesting structures of NTS1R that have been solved are the relevant cryo-EM structures of the SBI-553–NTS1R–GRK2–Gαq complex (8JPB, 8JPC^125^) reported by Duan, Xu, Yang, and collaborators in 2023,^125^ the complexes NTS1R–Go (PDB ID 8FN1 ^124^), SBI-553–NTS1R–Gq (PDB ID 8FMZ^124^), agonist SBI-553NTS1R–Go (PDB ID 8FN0 ^124^), reported by Krumm, Tenakin, Roth, and collaborators in 2023.^124^ IUPHAR/BPS Guide to Pharmacology^73^ and GPCRdb (gpcrdb.org)^78,126^ provide summaries of the primary and secondary coupling with G proteins that are currently recognized.

#### 2.2.5 Functional Precoupled Complexes

Interestingly, data suggest that, even when an agonist is not present, GPCRs commonly exist in preassembled complexes with transducers or effectors,^127–130^ reviewed by Lohse and collaborators 2012.^131^ Moreover, heterotrimeric G protein activation, rather than complete separation from the receptor,^132,133^ results often in structural reorganizations that lead each G protein subunit to interact with the corresponding effector^129^ while maintaining the whole signaling complex. Such examples were provided by (a) Bouvier and collaborators in 2006,^127^ who showed the formation of α_2A_ adrenergic receptor (α_2A_R)–Gi1αβ1γ2 complexes without the presence of an agonist using bioluminescence resonance energy transfer (BRET). (b) Lefkowitz, Bouvier, and collaborators, using single-particle electron microscopy in 2016, showed the presence of a megacomplex composed of a single agonist–GPCR−β-arr–G protein,^134^ and Lefkowitz, des Georges, and collaborators in 2019 ^128^ who solved the cryo-EM structure of an agonist–GPCR–G protein−β-arr megacomplex as the signaling megacomplex of an active chimeric β_2_AR coupled to a human G protein and bovine β-arr to the core and phosphorylated tail, respectively. This provided an example of a GPCR structure that can signal through a G protein from internalized compartments. (c) Ferré and collaborators, who suggested in 2018,^129^ using BRET and BiFC experiments that can be formed, beyond precoupled Gs–Gi–AC complexes, functional precoupled complexes consisting of heterotetramers of A_2A_R–dopamine D_2_ receptor coupled to their cognate Gs and Gi proteins and AC. The formation of these megacomplexes can be due to the random collision of signaling molecules in the plasma membrane, as shown for α_2A_R and Gi SMF microscopy with total internal reflection fluorescence (TIRF) imaging by Calebiro and collaborators in 2017 ^135^ instead of the rearrangement of precoupled units in a macromolecular complex.

An analysis of a large data set of MD simulations covering 60% of currently available GPCR structures by Selent and collaborators in 2025 ^136^ provides access to numerous previously unexplored GPCR conformational states and lipid interaction sites to hidden allosteric sites and even lateral lipid or ligand entrance gateways.

## 3 Multiple Conformations of Class A G Protein-Coupled Receptors

### 3.1 X-ray Crystallography, Cryo-EM

#### 3.1.1 G Protein-Coupled Fully Activated and Antagonist-Bound Inactive States

Class A GPCRs can exist in three different conformational states (active, inactive, intermediate-active) that can be interconverted for the activation/inactivation process according to a multistate, rheostat-like model,^14,137^ instead of a binary (on/off) switch model. The different conformational states responsible for the biased signaling and functional selectivity are allosterically exchanged through certain kinetic barriers, reviewed, for example, by Lefkowitz and collaborators in 2010,^37^ Christopoulos and collaborators in 2017,^42^ Thal, Christopoulos, and collaborators in 2018,^40^ Rajagopal, Lefkowitz, and collaborators in 2018.^38^

The structures of the ternary complexes of β_2_AR, A_2A_R β_1_AR, and μOR have been solved: (a) the X-ray structures of the agonist BI-167107−β_2_AR–Gαβγs^empty^ with Protein Databank Accession Identification Code (PDB ID) 3SN6 ^82^ reported by Kobilka, Sunahara, and collaborators in 2011;^82^ the agonist BI-167107−β_2_AR–Nb6B9 (Gs protein mimetic) complex (PDB ID 4LDE^138^) reported by Kobilka, Sunahara, Garcia, and collaborators in 2013,^138^ (b) the X-ray structure of the agonist NECA–A_2A_R–mini-Gs complex (PDB ID 5G53 ^56^) reported by Carpenter, Tate, and collaborators in 2016 ^56^ (Figure 1); the cryo-EM structure of the agonist adenosine–A_2A_R–Gαβγs^empty^ complex (PDB ID 6GDG^85^) reported by Tate and collaborators in 2018,^85^ (c) the cryo-EM structure of the agonist isoproterenol−β_1_AR–Gs^empty^ complex (PDB ID 7JJO^139^) reported by Huang, Liu, and collaborators in 2020,^139^ (d) the X-ray structure of the buprenorphine agonist BU72−μOR–Nb39 complex (G protein mimetic) reported by Kobilka and collaborators with cryo-EM in 2015 ^140^ (PDB ID 5C1M^140^) and reanalyzed by Munro in 2023 (PDB ID 8E0G^141^); the cryo-EM of the agonist peptide DAMGO−μOR–Gi^empty^ complex reported by Kobilka, Skiniotis, Manglik, and collaborators in 2018 ^142^ (PDB ID 6DDE^142^). The comparison of the structures of the complexes shows that the fully activated conformations of the class A GPCRs are similar. Indeed, the superposition of the NECA–A_2A_R–mini-Gs complex (PDB ID 5G53 ^56^) with the BI-167107−β_2_AR–Gs complex (PDB ID 3SN6 ^82^) reveals that the conformations of the corresponding GPCRs are strikingly very similar, as evidenced by the root-mean-square deviation (RMSD) in Cα carbons [RMSD­(Cα)] = 1.7 Å over 1239 Cα carbons. The intracellular segments of the receptors, including the significant outward displacement of the cytoplasmic end of TM6 during activation, are very well aligned.

Interestingly, the crystal structures of agonist isoproterenol−β_2_AR–Gs^GDP^ (PDB ID 6EG8 ^143^) or isoproterenol−β_2_AR–mini-Gs (PDB ID 6E67 ^143^) were reported by Liu, Kobilka, and collaborators in 2019.^143^ In these structures, β_2_AR adopts a fully activated conformation that matches the receptor conformation in crystal structure agonist BI-167107−β_2_AR–Gs^empty^ (PDB ID 3SN6 ^82^). The cryo-EM structures of the complexes between agonist morphine, fentanyl, or endomorphin with μOR–Gi^empty^ (PDB IDs 8EFQ, 8EF6, or 8F7R, respectively^144^) and other agonists were reported by Zhuang, Xu, and collaborators in 2022.^144^

The pivotal outward movement of TM6 generates a cavity in the core of the cytoplasmic region of the receptor formed by ICL2, TM3, TM5, and TM6, in which the C-end of Gαs can insert. The comparison of inactive and active structures of β_2_AR is informative. These are, for example, the X-ray structure of the inactive state of β_2_AR in complex with the inverse agonist carazolol (PDB ID 2RH1 ^62^) reported by Stevens, Kobilka, and collaborators in 2007;^62^ the inverse agonist ICI 118,551 (PDB ID 3NY8 ^67^) and antagonist alprenolol (PDB ID 3NYA^67^) reported by Stevens and collaborators in 2010;^67^ the inverse agonist carazolol (PDB ID 5D5B^145^) reported by Caffrey, Wang, and collaborators in 2016,^145^ and the β_2_AR-Gs^empy^ in the active complex PDB ID 3SN6.^82^ The comparison of the structures showed the conformational changes of the receptor in its interface with Gs. Briefly, ICL2 adopts an α-helix conformation; in the extension of TM5, the N-end of ICL3 forms an α-helix, and TM6 is extended outward. The ICL2, and especially F139^ICL2^ (residue 34.51 in the numbering scheme in GPCRdb^78,126^ ) fits in the hydrophobic site formed by the αN/β1 hinge, β2/β3 loop, and F376 at the Cα5 helix of Gαs. This is the critical step to induce GDP release, as also shown by Chung, Kobilka, Lodowski, and collaborators.^26^ This structural rearrangement is made easy because of the flexibility of the C-terminal Cα5 helix following reduction of its interactions with the Ras domain.

Such movements are observed by comparison also of the inactive A_2A_R (in its complex with inverse agonist ZM241385) reported by Stevens and collaborators in 2008 (PDB ID 3EML^61^) and by Stevens, Cherezov, and collaborators (PDB ID 4EIY^57^) with the structure of the fully activated A_2A_R (PDB ID 5G53 ^56^) shown in Figure 1, the inactive β_1_AR in the complex of β_1_AR–T4L with the high-affinity antagonist cyanopindolol (PDB ID 2VT4 ^66^) reported by Schertler, Tate, and collaborators in 2008,^66^ with the structure of the fully activated β_1_AR in isoproterenol−β_1_AR–Gs^empty^ complex (PDB ID 7JJO^139^), the crystal structure of the inactive μOR in the complex morphinan antagonist−μOR (PDB ID 4DKL^146^) reported by Granier, Kobilka, and collaborators in 2012,^146^ with the structure of the fully activated μOR in the complex agonist BU72−μOR–Nb39 (PDB ID 5C1M^140^). Note that T4-lysozyme (T4L) fused into the ICL3 is a GPCR modification widely used in crystal structure determination. Interestingly, the R^3.50^-E^6.30^ “ionic lock” interaction that is observed in the inactive state of A_2A_R^61^ is not present in the X-ray structures of the inactive conformations of β_2_AR and β_1_AR^63–67^ and of μOR^146^ (see relevant discussion after Figure 1). Instead, residue R^3.50^ in the crystal structures of the inactive state of β_2_AR or μOR forms a salt bridge with the adjacent D^3.49^ of the DRY sequence.

Experimental structures have shown that in the fully activated conformation, the intracellular outward shift of TM6 from the intracellular TM7, which is one of the key determinants for the interaction of class A GPCRs with G proteins and arrs, can vary between GPCRs in magnitude and relative orientation compared to the location it has in the inactive state.^3,25,63,147,148^ The activation level and the magnitude of the overall motion are reflected by the magnitude of the conformational changes in CWxP (C^6.47^W^6.48^P^6.50^), PIF (P^5.50^I^3.40^F^6.44^), NPxxY (N^7.49^P^7.50^xxY^7.53^), and “ionic lock” microswitches. Comparatively, a large outward shift of TM6, as the distance between the Cα atoms of Thr224^6.26^ from the inactive to the active conformation has been measured in class A GPCRs bound to an agonist and a G protein, for example, ∼6 Å in RhoR,^62^ and ∼14–18 Å in β_2_AR,^62,82^ and A_2A_R.^56,61^

#### 3.1.2 Multiple Transducer-Coupled States

The β_2_AR, β_1_AR, and A_2A_R bind with high selectivity to Gs, which is the G protein that stimulates AC. The β_2_ and β_1_ adrenergic receptors can also couple to Gi proteins that inhibit AC, but the coupling preference is much smaller. β_2_AR can also couple to Gq proteins. The μOR is primarily coupled to Gi/o proteins that inhibit AC. All these class A GPCRs also couple to β-arrs following receptor phosphorylation. Tate and collaborators reported in 2020 ^105^ the cryo-EM structure of the agonist formoterol−β_1_AR−β-arr1 complex (PDB ID 6TKO^105^). In structures of signaling complexes of class A GPCRs that couple to a Gi protein, the outward movement of TM6 is smaller compared to Gs and Gq/11 complexes because of the smaller size of the Gαi protein binding pocket in the cytoplasmic core of the receptor compared to the Gαs and Gαq proteins. This is shown, for example, by comparison of the X-ray structure of BI-167107−β_2_AR–Gs complex (PDB ID 3SN6 ^82^), and the cryo-EM structure of the agonist peptide DAMGO−μOR–Gi1 complex (PDB ID 6DDE^149^) reported by Kobilka, Skiniotis, Manglik, and collaborators in 2018.^149^

#### 3.1.3 Agonist-Only-Bound States

Class A GPCRs in complex with only full agonists (without G or βarr protein) adopt an intermediate active conformation, which might be a preactive conformation in the activation pathway. Τhe structure of class A GPCRs in such transient conformations has not yet been thoroughly characterized, despite its importance in understanding the different interactions with diverse signal transducers (G proteins or arrs) that initiate the intracellular signaling after an extracellular stimulus or agonist binding. The detailed activation mechanism by which the binding of the agonist at the extracellular region of the GPCR is transmitted allosterically at the intracellular region, which opens to bind a G protein, is likely to differ between different categories of class A GPCRs and even for distinct subtypes of the same family.

##### 3.1.3.1 A_2A_R: Intermediate Active and Inactive-like Conformations

The X-ray structures of agonist-only bound A_2A_R (without Gs protein or Nb as Gs mimetic), e.g., agonist NECA (PDB ID 2YDV^150^) or adenosine (PDB ID 2YDO^150^) were reported by Tate and collaborators in 2011.^150^ Relevant experimental structures have revealed that the transformation of A_2A_R from an inactive structure (PDB ID 3EML^61^) to the intermediate active (PDB ID 2YDV^150^) and then to the fully active conformation (PDB ID 5G53 ^56^) is described by an outward tilt of the cytoplasmic TM6 from the receptor core by ∼40° rotation of TM6 about F282^6.44^ and ∼4 Å between the Cα atoms of Thr224^6.26^ and an additional ∼14 Å as compared to the corresponding conformations of A_2A_R.

When the structures of the antagonist–A_2A_R (PDB ID 3EML^61^), agonist–A_2A_R complexes (PDB ID 2YDV^150^) are compared with the ternary A_2A_R–agonist–mini-Gs complex (PDB ID 5G53 ^56^), it seems that the extracellular OBS exhibits a highly dynamic connection to the intracellular surface in the intermediate active state. The same in-principle conformational perturbation in A_2A_R is observed when considering both the two changes from antagonist-bound to agonist-bound or from antagonist-bound to agonist-bound–Gs A_2A_R structures. Therefore, the intermediate active A_2A_R conformation has a similar conformation in CWxP, PIF, and NPxxY motifs to the fully active bound conformation. The results for A_2A_R support a model in which agonist binding is adequate to populate a conformation that resembles the fully active state, i.e., it is active-like. Thus, a strong allosteric connection in A_2A_R exists between the conformation of the intracellular region where the G protein binds and the agonist-bound OBS region.^40,42,44^ This connection must be assumed for the receptor to bind the Gs protein, which triggers the signaling cascade. This allosteric network connection is facilitated by the structurally flexible “toggle switch” residue W^6.48^ motion that causes the outward movement and rotation of TM6, which initiates the conformational changes for receptor activation.

While in all previous structures of A_2A_R in complex with only agonist (PDB ID 2YDV^150^), the receptor lies in a conformation that is very similar, as regards the CWxP, PIF, and NPxxY motifs, to the fully activated state (PDB ID 5G53 ^56^). In the X-ray structure of A_2A_R in complex with only partial agonists LUF5834 (PDB ID 8RLN^151^) or LUF5833 (PDB ID 7ARO^152^), the receptor adopts an inactive-like conformation, as was shown by Ijzerman and collaborators in 2021 ^152^ or Müller and collaborators in 2024.^151^ However, in ensembles of A_2A_R, partial agonists bind and stabilize intermediate conformations in the active region of the conformational space. This has been shown with ^19^F solution NMR in micelles by Prosser, Ernst, and collaborators in 2016 ^68^ or lipid nanodiscs by Prosser, Sljoka, and collaborators in 2021;^153^ smFRET in micelles by Gradinaru, Prosser, Ye, and collaborators in 2021;^154^ solution ^15^N NMR in micelles by Eddy and collaborators in 2021,^155^ and 2022 ^156^ and solution ^19^F NMR in micelles by Eddy and collaborators in 2024;^157^ ^19^F solution NMR in micelles by Ye and collaborators in 2023 ^55^ and Ye, Cheng, Miao, and collaborators in 2025 ^80^ (discussed afterward with NMR findings). Thus, while partial agonists can bind both active and inactive conformations, they have a higher binding affinity for active conformations. However, full agonists of A_2A_R bind solely to the active conformation, likely because of conformation selection. This might be a mechanism due to a partial agonism that works with several GPCRs, as suggested in ref.^151^ Interestingly, in PDB ID 8RLN,^151^ 3-cyano group is hydrogen-bonded with N253^6.55^, the 2-amino group is hydrogen-bonded through water bridges with His264^ECL3^ and E169^ECL2^, and the 5-cyano group is hydrogen-bonded through water with His278^7.42^. This has also been observed with a series of antagonists having a similar cyano group that bind human A_3_ adenosine receptor by Kolocouris, Ladds, Pouli, and collaborators in 2022 based on alchemical relative binding free energy calculations, MD simulations, kinetic BRET-based binding experiments, functional assays, and mutagenesis experiments.^158^ Interestingly, Lane and collaborators in 2012 ^159^ suggested that alanine mutation of N253^6.55^ did not change the affinity of LUF5834 but removed its agonist activity. In ref 159, it was speculated that N253^6.55^ is hydrogen-bonded with the exocyclic amino group of the pyridine ring, and therefore, the results of the mutagenesis experiments suggested an alternative binding profile. However, the X-ray structure revealed^151^ that the 3-cyano group was engaged in hydrogen bonding with N253^6.55^, and even in the presence of residue N253^6.55^, the 2-amino group can still stabilize the ligand through hydrogen bonding interactions.

##### 3.1.3.2 β_2_AR, β_1_AR: Inactive-like Conformations

In structures of β_2_AR that are bound only to agonists, for example, in the X-ray structure of β_2_AR with the covalent full agonist FAUC50 (PDB ID 3PDS^160^) reported by Kobilka, Gmeiner, Caffrey, and collaborators in 2011,^160^ the receptor adopts an intermediate-active conformation that closely resembles the inactive conformation, for example, has a similar PIF conformation and lacks an outward shift of cytoplasmic TM6. Additionally, in the structure of complexes of β_1_AR with agonists or partial agonists (PDB IDs 2Y02, 2Y03, 2Y04 ^161^), reported by Tate and collaborators in 2011,^161^ PIF adopts an inactive-like conformation. Similarly, to A_2A_R, in an ensemble of β_2_AR, a partial agonist binds and stabilizes an active intermediate conformation, as was shown by Shimada and collaborators in 2020 ^162^ with ^15^N solution NMR (discussed afterward with NMR findings).

The conformation of the PIF motif of the inactive state is present in the X-ray structures of the β_2_AR with the inverse agonist ICI 118,551 (PDB ID 3NY8 ^67^) and carazolol (PDB ID 5D5B^145^) or the antagonist alprenolol (PDB ID 3NYA^67^) and in the X-ray structure of the β_1_AR in complex with the high-affinity antagonist cyanopindolol (PDB ID 2VT4 ^66^). In contrast, as mentioned previously in the complex of A_2A_R with only an agonist (PDB IDs 2YDV, 2YDO^150^), the receptor adopts an intermediate active PIF conformation, which is like the fully activated conformation. Thus, in contrast to A_2A_R, β_2_AR, or β_1_AR showed a weak allosteric connection between the agonist’s OBS and the cytoplasmic ends of TM5 and TM6 that must be engaged with the G protein for activation.

#### 3.1.4 Intermediate Active GPCR States

It was previously discussed that in the X-ray structures of full agonist adenosine or NECA-bound A_2A_R complexes (PDB IDs 2YDO or 2YDV, respectively^150^), the receptor adopts an intermediate active conformation. Remarkably, it was also shown using cryo-EM, ^19^F NMR, and enhanced sampling MD simulations that A_2A_R exists in an adenosine–A_2A_R–mini-Gαsβγ complex with R291^7.56^A mutation in an intermediate active conformation (see PDB IDs 9EE8, 9EE9, 9EEA^80^), since this mutation prevents the receptor from adopting the fully activated state.^80^ According to the functional assays in ref 80 in this intermediate conformation, R291^7.56^A A_2A_R has a limited rate of exchange of GDP to GTP, i.e., Gαs cannot adopt its fully activated conformation.

## 4 NMR, DEER, SMF, Biomolecular Simulations

### 4.1 A_2A_ Adenosine Receptor

#### 4.1.1 Conformations of Cytoplasmic TM6

##### 4.1.1.1 Exploration of the Conformational Equilibrium: Characterization of the Conformers

**a. Studies in Micelles**

The conformational changes of A_2A_R in mixed neopentyl glycol-3 (MNG-3)/cholesteryl hemisuccinate (CHS) micelles were investigated by Shimada and collaborators in 2020 ^163^ using [^13^C,^1^H] solution NMR spectroscopy with ^13^C-Met labeled receptor in the cytoplasmic region by applying the following mutations: I106^3.54^ M, A232^6.34^ M, V239^6.41^ M, I292^8.47^ M. Interestingly, at least one class A GPCR has a methionine residue at all positions: 3.54, 6.34, 6.41, and 8.47. In the presence of the inverse agonist (ZM241385), the chemical shifts of these Met resonances of A_2A_R were significantly different from those in the presence of the full agonist (NECA), indicating that the activation of A_2A_R causes a significant conformational change. While in the presence of a partial agonist (LUF5834), an equilibrium exists between the active state (including multiple species) and the inactive state; the addition of a full agonist shifts the population of the conformations in the direction of the active state.^164^

Prosser and collaborators applied solution ^19^F NMR spectroscopy in 2016–2018,^68,165,166^ to V229^6.31^C-labeled A_2A_R in mixed neopentyl glycol-3 (MNG-3)/CHS micelles to detect conformational changes at the cytoplasmic TM6. This research^68^ showed in apo-A_2A_R the equilibrium of the inactive conformations **S1** ^**TM6**^ and **S2** ^**TM6**^, the active intermediates **I1** ^**TM6**^, **I2** ^**TM6**^, and the **A** ^**TM6**^ that correspond to the fully activated conformation, see Figure 3. These conformations were characterized based on their chemical shifts in the active region and through an increase in population by the addition of an agonist and a G protein-derived peptide (Gαs peptide).^80,167^ The results suggested that even in the inactive state, class A GPCRs exhibit conformational heterogeneity.

**Figure 3:**
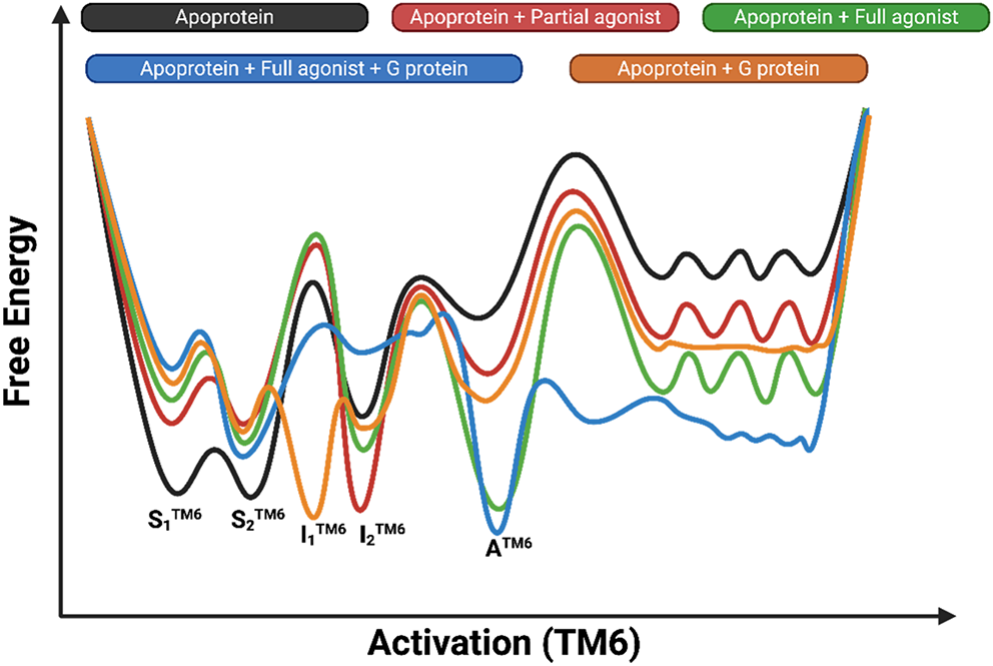
Representation of the free energy profile of the A_2A_R with or without agonist and G protein along a reaction coordinate that describes perturbation of TM6. The landscape describes an ensemble of conformations that correspond to the free energy minima that the receptor occupies. These minima include inactive and active states identified by solution NMR, SMF, DEER, and smFRET studies complemented by simulations. The free energy landscape of the A_2A_R includes the two inactive conformations (**S1** ^**TM6**^ and **S2** ^**TM6**^) that can be converted for receptor activation to the active intermediates **I1** ^**TM6**^, **I2** ^**TM**6^, and then to the fully activated conformation **A** ^**TM6**^. **I2** ^**TM**6^ and **A** ^**TM6**^ conformations have been characterized as low-efficacy and high-efficacy activation states, reinforced by full agonist (green line) and partial agonist (red line), respectively. Additionally, **I2** ^**TM**6^ and **A** ^**TM6**^ conformations correspond to noncognate Go and cognate Gs complexes, nucleotide-free G protein activation states. The populations of the TM6 activation states (**I1** ^**TM6**^, **I2** ^**TM**6^, **A** ^**TM**6^) and TM7 activation states (**I1** ^**TM7**^, **I2** ^**TM7**^, **A** ^**TM7**^) may be correlated, albeit they are not identical (figure inspired by ref 153).

Conformations **S1** ^**TM6**^ and **S2** ^**TM6**^ were considered with a formed and broken “ionic lock” interaction,^68^ respectively (Figure 4). This is observed in the crystal structures of complexes of A_2A_R with antagonist/inverse agonist ZM241385, correspondingly in the ZM241385–A_2A_R-StaR2 complex (PBD ID 3PWH 161) and in the ZM241385–A_2A_R–T4L complex (PBD ID 3EML^61^), respectively. However, as was noted by Marshall and collaborators in 2011,^168^ the T4 lysozyme fusion in ICL3 may cause an outward movement and rotation in TM6 in structure PBD ID 3PWH,^168^ which is why the “ionic lock” may be absent in the A_2A_R–T4L structure. Ye and collaborators in 2023,^55^ using solution ^19^F NMR and MD simulations of A_2A_R labeled at cytoplasmic TM6 in MNG-3/CHS micelles complemented by MD simulations, showed that **S1** ^**TM6**^ conformation is stabilized not only by the DR^3.50^Y-E^6.30^ “ionic lock” interaction but also cation-π interactions (R291^7.56^-H230^6.32^ and R293^8.48^-H230^6.32^ interactions) are present, with TM3, TM6, and TM7/H8 closely clustered together (see also previous discussion on the “microswitch” motif network). **S1** ^**TM6**^ conformation bears the DR^3.50^Y-E^6.30^ “ionic lock” interaction and the cation-π contacts. As activation proceeds, the **S1** ^**TM6**^ state transitions to the **S2** ^**TM6**^ state, where the ionic lock (DR^3.50^Y-E^6.30^) is broken; only the R291^7.56^-H230^6.32^ is required for the stabilization of the **I2** ^**TM6**^ conformation. The mutation R291^7.56^A traps A_2A_R in conformation **I2** ^**TM6**^, preventing the receptor from adopting the fully activated conformation **A** ^**TM6**^;^55^ R293^8.48^ is required for maintaining the **I2** ^**TM6**^ conformation, suggesting that its further disengagement with H230^6.32^ will lead the receptor toward the complete opening of the G protein binding cavity in the fully activated-like conformation **A** ^**TM6**^.^55^

**Figure 4:**
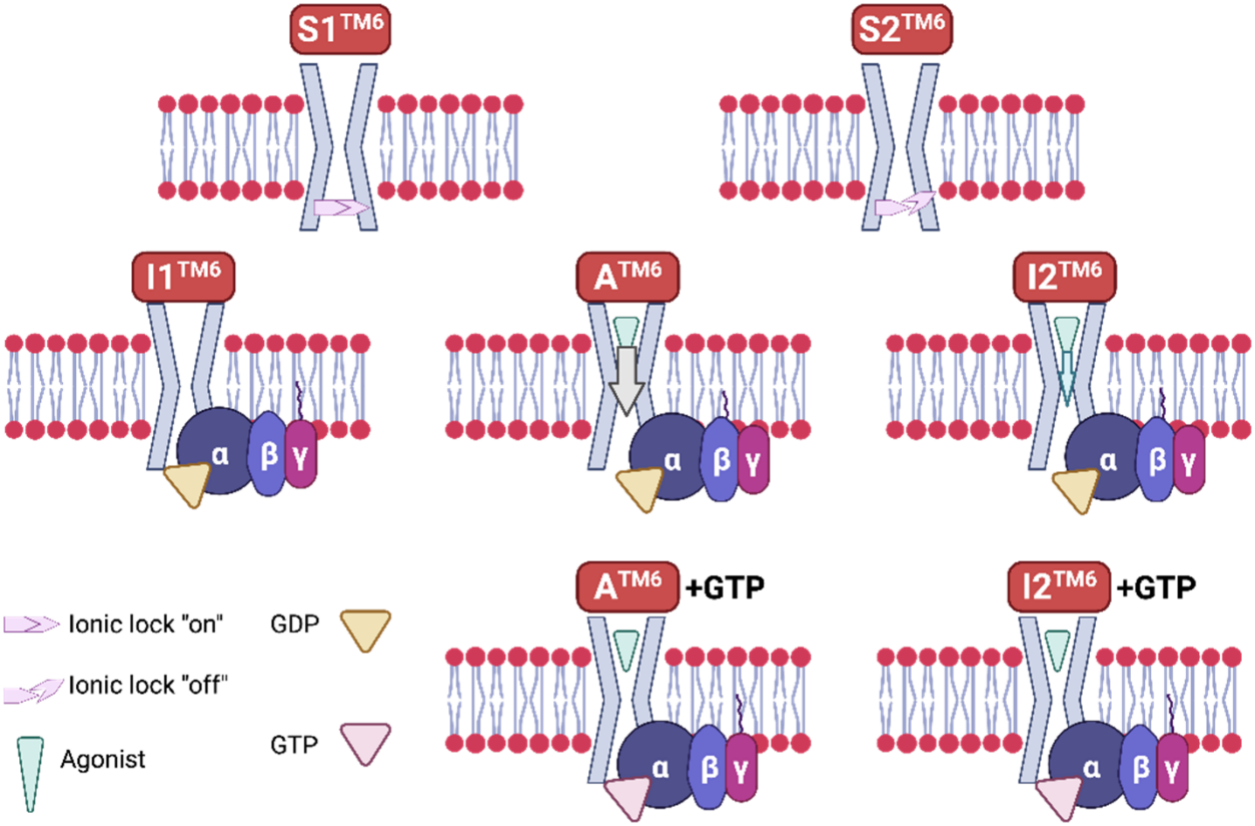
Graphic of the conformational ensemble describing a class A GPCR (e.g., A_2A_R with Gs protein) activation pathway, which can include many transient conformations based on labels of cytosolic TM6. A subset of 5 conformational states, **S1** ^**ΤM6**^, **S2** ^**ΤM6**^, **I1** ^**ΤM6**^, **I2** ^**ΤM6**^, **A** ^**ΤM6**^, and the corresponding complexes of **I1** ^**ΤM6**^, **I2** ^**ΤM6**^, **A** ^**ΤM6**^ can be formed after G addition, as was observed in solution NMR and smFRET studies in micelles or lipid nanodiscs for A_2A_R.^55,68,80,153,167^ A transient stable agonist–GPCR–G^GTP^ complex must also be involved, although not observed in the experimental work. In the graphic are shown five important functional states: two inactive states, **S1** ^**ΤM6**^ and **S2** ^**ΤM6**^, that are distinguished by an “ionic lock” formed and a broken “ionic lock” DR^3.50^Y-E^6.30^ interaction, the active intermediate conformation **I1** ^**ΤM6**^ and the two conformations **I2** ^**ΤM6**^ and **A** ^**ΤM6**^ in the active region of the TM6 conformational space. There is no interaction between the Gαβγ^GDP^ and GPCR in the inactive conformations **S1** ^**ΤM6**^, **S2** ^**ΤM6**^. A combination of GTP hydrolysis rates of apo-form, partial agonist-, and full agonist-bound class A GPCR and the corresponding solution ^19^F NMR spectra, e.g., for A_2A_R in ref 153, showed that the active conformations, **I2** ^**ΤM6**^ and **A** ^**ΤM6**^ bind G^GDP^ and release the nucleotide by subsequently forming the more stable agonist–GPCR–G^empty^. **A** ^**ΤM6**^ conformation is stabilized more by a full agonist and is more efficacious than the **I2** ^**ΤM6**^ conformation since the latter is stabilized preferentially by a partial agonist. I**2** ^**ΤM6**^, **A** ^**ΤM6**^ conformations are significantly populated in the presence of an agonist and Gαβγs protein forming an agonist–GPCR–G^empty^ complex (which is particularly stabilized after releasing of GDP from agonist–GPCR–G^GDP^). Thus, a partial agonist can bind to GPCR, changing the receptor’s conformation to **I2** ^**ΤM6**^ and facilitating in the presence of G protein Cα5, insertion into GPCR’s binding site while AHD opens the GDP binding site of Gα (see Figure 2). **A** ^**ΤM6**^ conformation triggers an additional opening of the cytoplasmic region in GPCR, which causes, in the presence of G protein, stronger binding of Cα5 of Gαβγ to the receptor, resulting in GDP/GTP exchange. After GTP binds to Gαβγ, the Gαβγ uncouples from the receptor and dissociates into the Gα and Gβγ as described in Figure 2. **A** ^**TM6**^ for A_2A_R may correspond to the fully activated conformation in the NECA–A_2A_R–mini-Gs complex with PDB ID 5G53 ^56^ determined by X-ray crystallography. It was shown that **I2** ^**TM6**^ for A_2A_R has the conformation determined for adenosine–A_2A_R–mini-Gαsβγ complex^80^ with receptor bearing the mutation R291^7.56^A (PDB IDs 9EE8, 9EE9, 9EEA^80^). **I1** ^**ΤM6**^ conformation corresponds to the complex GPCR–G (figure inspired by ref 153).

In the presence of a full agonist, the solution ^19^F NMR spectrum showed the presence of conformations **A** ^**TM6**^ and **I2** ^**TM6**^ in the active region of the TM6 conformation space, but also the inactive **S2** ^**TM6**^ conformation; in the presence of antagonist/inverse agonist ZM241385, the spectrum contained the inactive conformations **S1** ^**TM6**^ and **S2** ^**TM6**^, the conformation **I2** ^**TM6**^ and a minor population of conformation **A** ^**TM6**^ (Figure 3).^68^ Compared to the apo-form, the addition of a full agonist (e.g., NECA) stabilizes **A** ^**TM6**^ conformation with a similar population, while the addition of a partial agonist (e.g., LUF5834) significantly increases the population of active intermediate **I2** ^**TM6**^ shown.^68^

The population of the inactive conformations **S1** ^**TM6**^ and **S2** ^**TM6**^ of the apo-form was increased in the presence of the Na^+^ cation in agreement with its allosteric stabilization of the inactive state, as was shown by Prosser and collaborators in 2018 using ^19^F solution NMR of A_2A_R in MNG-3/CHS micelles.^153^ This was also shown in the same work^153^ using ^23^Na NMR. This effect of Na^+^ ions on class A GPCRs conformation has been shown using nMS by Robinson and collaborators in 2021.^55^ In contrast, the presence of the divalent cations shifted the equilibrium in the apo-form toward the active state (**I2** ^**TM6**^ and **A** ^**TM6**^); the positive allosteric effects of Ca^2+^ or Mg^2+^ are more pronounced when an agonist and a mini-Gαs (Gαs-protein-derived peptide) were present. High concentrations of divalent cations allosterically drive the opening of the G-protein-binding cavity by connecting certain extracellular acidic residues, bringing TM5 and TM6 together at the extracellular surface according to MD simulations.^159^ Understanding cation allostery can improve our knowledge of GPCR regulation in the cellular environment and help design allosteric drugs.

Multiscale MD simulations of A_2A_R in a membrane-like phospholipid bilayer and detergent micelles, performed by Vaidehi and collaborators in 2020,^137^ supported the presence of three different general conformational states (active, inactive, intermediate-active) detected experimentally as static structures with X-ray or cryo-EM or in equilibrium using NMR. McCammon, Miao, and collaborators have developed Gaussian-accelerated MD (GaMD)-based simulation methods for describing the conformational space of class A GPCRs.^169–174^

Gradinaru, Prosser, Ye, and collaborators applied in 2021 ^154^ single-molecule FRET (smFRET) to A_2A_R in MNG-3/CHS micelles to resolve active and inactive states via the separation between TM4 and TM6 through specific sites in TM4 and TM6, that were labeled with a donor–acceptor dye pair (AF488-AF647 dye pair) at residues T119 and Q226^6.28^, respectively; also the dynamics of TM6 were followed by labeling the cytoplasmic end of TM6 at V229C^6.31^ with an environment-sensitive dye (BODIPY-FL). According to the magnitude of smFRET signals and the measured range of distances between labeled cytoplasmic TM4 and TM6 in MNG-3/CHS micelles,^154^ and in agreement with previous ^19^F solution NMR studies,^68^ it was suggested that the partial agonist might stabilize a conformation that can correspond to the active intermediate **I2** ^**TM6**^ stabilized by partial agonist LUF5834 in Gs signaling, based on a cyclic AMP (cAMP) assay, with a TM6-TM4 separation distance with values corresponding between the fully activated-like conformation **A** ^**TM6**^ (open in cytoplasmic region exhibiting high-FRET) and inactive state with a closed in cytoplasmic region, exhibiting low-FRET. The active intermediate conformation **I2** ^**TM6**^ of A_2A_R stabilized by a partial agonist, e.g., LUF5834, is different from the active-like conformation **A** ^**TM6**^ stabilized by a full agonist, e.g., NECA (Figure 4). It was suggested^154^ that **A** ^**TM6**^ conformation is similar to the fully activated conformation observed in the X-ray structure of agonist NECA–A_2A_R–mini-Gs complex (PDB ID 5G53 ^56^) or the cryo-EM adenosine–A_2A_R–Gαβγs^empty^ complex (PDB ID 6GDG^85^). Similarly, it was also suggested that conformation **I2** ^**TM6**^ might be like the intermediate-active conformation of A_2A_R observed in the crystal structure of agonist NECA–A_2A_R (PDB ID 2YDV^150^). **I2** ^**TM6**^ stabilized by the partial agonist LUF5834 may also be involved in β-arr coupling. Indeed, Franco and collaborators in 2020,^175^ using functional assays, FRET-based binding assays, and MD simulations, showed that LUF5834 was as efficient as full agonists CGS21680 and adenosine in β-arr recruitment. Interestingly, agonists PSB0777 and NECA recruit β-arr more efficiently compared to agonists adenosine and CGS21680, but it was not feasible to provide details of these preferences through MD simulations.^175^

Eddy and collaborators in 2021 ^155^ used singly mutated-A_2A_R at cytoplasmic TM5, TM6, or TM7 ends with tryptophan residues as reporter groups, enabling the observation of receptor responses to bound drugs of different efficacy, and a mini-Gas consisting of a 21-residue polypeptide from the Gαs carboxy terminus. The corresponding F201^5.62^W or K233^6.35^W or Y290^7.55^W A_2A_R was reconstituted in lauryl maltose neopentyl glycol (LMNG)/CHS micelles that, compared to dodecyl-β-D-maltoside (DDM)/CHS micelles, provided higher resolution [^15^N,^1^H] solution NMR spectra. The overall protein fold and ligand binding activity of the three A_2A_R variants were highly similar to those of the native protein. While a single ^15^N–^1^H signal for each of these tryptophans was measured for the complexes with antagonists, two signals were observed for a complex of K233^6.35^W A_2A_R with an agonist (e.g., UK432097). However, no ^15^N–^1^H signals were plotted for the complexes of agonist UK432097 with F201^5.62^W or Y290^7.55^W A_2A_R. Importantly, when the mini-Gαs was added to the UK432097–A_2A_R complex, one peak for W233^6.35^ was observed in comparison to the two peaks in the agonist UK432097–A_2A_R complex, showing the impact of the allosteric coupling in GPCR signaling complexes. While endogenous tryptophans at the extracellular surface did not exhibit any signal after the mini-Gαs peptide addition, the endogenous tryptophans at the intracellular surface showed an NMR signal. This agreed with X-ray structures of binary and ternary complexes of A_2A_R involving an agonist and a mini-Gαs, where conformational changes of A_2A_R in the ternary agonist NECA–A_2A_R–mini-Gαs complex (PDB ID 5G53 ^56^) compared to the agonist–A_2A_R complex (PDB ID 2YDV^150^) were observed in the intracellular region but not in the extracellular region.

Eddy and collaborators in 2021 ^155^ and in 2022 ^156^ obtained [^15^N,^1^H] solution NMR spectra of A_2A_R in LMNG/CHS micelles, focusing on signals from ^15^N–^1^H signals of W246^6.48^ indole. It was shown^155^ that partial agonists, compared to full agonists, induce a structure with a different conformation of cytosolic TM7 and tryptophan “toggle switch” (W^6.48^). The conformation and chemical shift of W^6.48^ are affected by the orientation of the neighbor F^6.44^, suggesting different conformations in the conserved PIF motif. In ref 156, it was shown that the critical residue W246^6.48^ for activation of the receptor showed large fluctuations in the ternary complex agonist NECA–A_2A_R–mini-Gs, suggesting the allosteric connection between the bound Gs protein and the OBS, which, after drug-binding perturbation, causes the structural plasticity of the “toggle switch” W246^6.48^. In the same work,^156^ it was shown that in the ternary and agonist-only bound complexes, the conformation of A_2A_R is almost similar, with only subtle changes at the receptor cytoplasmic surface, suggesting the conformational selection of the active conformation only after agonist binding. This has also been revealed in the crystal and cryo-EM structures of hA_2A_R in which the intermediate active conformation of the agonist-only bound structure (PDB IDs 2YDV, 2YDO^150^) matches the fully activated structure (PDB IDs 5G53 ^56^ and 6GDG^85^) in all CWxP, PIF, and NPxxY activation motifs.

To comprehend the conformational changes that occur during GPCR activation, in continuation of the work in ref 55, Ye, Cheng, Miao, and collaborators in 2025 ^80^ applied solution ^19^F NMR to labeled A_2A_R at V229^6.31^C in the cytoplasmic half of TM6 in LMNG micelles without and with Gαβγs or mini-Gαsβγ, as well as GTP hydrolysis and nucleotide exchange assays that used BODIPY-FL–GTP and BODIPY-FL–GDP in LMNG micelles. In the reported cryo-EM structure of the adenosine–A_2A_R–mini-Gαsβγ complex^80^ with A_2A_R bearing the mutation R291^7.56^A (PDB IDs 9EE8, 9EE9, 9EEA^80^) the mutant R291^7.56^A A_2A_R adopts the **I2** ^**TM6**^ conformation, which was also studied with GaMD simulations (Figures 3 and 4). A_2A_R with mutation R291^7.56^A was isolated to the activation intermediate adenosine–A_2A_R-Gαβγs complex in the **I2** ^**ΤM6**^ conformation that permits the complexation of Gαs^GDP^. This receptor with **I2** ^**ΤM6**^ conformation can bind GDP but has a limited rate for the critical GDP/GTP exchange.

**b. Studies in Lipid Bilayers**

**A**_**2A**_ **R Conformation**

Solution ^19^F NMR spectroscopy was applied to V229^6.31^C-labeled A_2A_R, also in 1-palmitoyl-2-oleoyl-*sn*-glycero-3-phosphocholine (POPC)/1-palmitoyl-2-oleoyl-*sn*-glycero-3-phosphoglycerol (POPG) nanodiscs by Prosser, Sljoka, and collaborators in 2021.^153^ It is noted that lipid nanodiscs are reconstituted high-density lipoproteins (rHDLs).^164,176^ The ^19^F solution NMR spectra were obtained with or without ligands, Gs heterotrimer, or GDP at saturating concentrations.^153^ Similar to the spectra in micelles,^68^ the spectra in lipid nanodiscs revealed the presence of active conformations **A** ^**TM6**^, **I2** ^**TM6**^ and inactive conformations **S1** ^**TM6**^, **S2** ^**TM6**^ (Figures 3 and 4); in the spectra after full agonist addition, the **A** ^**TM6**^ conformation was stabilized and after partial agonist (e.g., LUF5834) the **I2** ^**TM6**^ conformation was stabilized, while a minor population of inactive conformation (possibly **S2** ^**TM6**^) was also observed. Interestingly, in the ^19^F solution NMR study of A_2A_R in POPC/POPG nanodiscs, the active state of A_2A_R exhibited 50% of the population.^153^ Based on the signal intensity, it was shown that conformations **A** ^**TM6**^, **I2** ^**TM6**^ form agonist–A_2A_R–Gαβγs^empty^ complexes by the addition of Gs^empty^.

The addition of Gαβγs^GDP^ in agonist-containing A_2A_R sample causes a shift of the population toward the active states. Both **A** ^**TM6**^ and **I2** ^**TM6**^ were implicated in receptor activation, according to a combination of GTP hydrolysis rates of apo-form, partial agonist-, and full agonist-bound A_2A_R and the corresponding solution ^19^F NMR spectra.^153^ The GTP hydrolysis-based assays showed that the **A** ^**ΤM6**^ and **I2** ^**ΤM6**^ conformations promote GDP release by subsequently forming the more stable agonist–GPCR–Gs^empty^ conformation; thus, agonist–GPCR–Gs^GDP^ is an intermediate before the formation of the agonist–GPCR–Gs^empty^ complex. This was also shown with MD simulations of the A_2A_R activation path, performed by Vaidehi and collaborators in 2019.^177^ When Gαs^GDP^ was added to the apo-receptor, the population of **I1** ^**TM6**^ conformation was also increased, suggesting the formation of the precoupled A_2A_R–Gαs^GDP^ complex. It was also suggested^153^ the Gβγ subunit is essential for transmitting the efficacy of the ligand from the receptor to the GDP binding site in Gα. Therefore, while investigating allosteric mechanisms linked to G protein activation mediated by the receptor, research must be performed on the Gβγ subunit in addition to the Gα subunit.

Interestingly, a metadynamics simulation study of the A_2A_R in POPC/cholesterol bilayers was reported by Limongelli and collaborators in 2024,^58^ with two collective variables used to describe TM6 rotation and translation. The purpose of the study^58^ was to describe the conformational landscape in the activation mechanism, using as starting structures the apo-A_2A_R and antagonist- or agonist-bound states. In addition to the “standard” free energy minima, including the inactive conformations (**S1** ^**TM6**^ and **S2** ^**TM6**^) and active conformations (**A** ^**TM6**^ and **I2** ^**TM6**^), an additional “pseudo-active” intermediate conformation, **Ix** ^**TM6**^, was found, characterized by an “activating ionic lock” formed also in **A** ^**TM6**^ and **I2** ^**TM6**^ conformations. This “activating ionic” R^5.66^-E^6.30^ interaction has been observed in the X-ray structure of the active conformation of RhoR (PDB IDs 3CAP^178^ and 3DQB^179^) reported by Ernst, Choe, Hofmann, and collaborators in 2008,^178,179^ having, however, the mutation R^5.66^K. In the experimental structures of the fully activated conformations of A_2A_R (PDB IDs 5G53 ^56^ and 6GDG^85^) and also in the cryo-EM structures of adenosine–hA_1_R–Gi complex (PDB ID 7LD3 174) reported by Christopoulos, Imlach, Glukhova, and collaborators in 2021,^180^ and the NECA–hA_2B_R–Gs complex (PDB ID 8HDP^181^) reported by Jiang, Xie, Xu, and collaborators in 2022,^181^ the ICL3 (close to E^6.30^) that connects TM5 and TM6 was unresolved. The conformation **Ix** ^**TM6**^ can serve as an intermediate (e.g., **I1** ^**TM6**^) during activation, facilitating coupling with the Gs protein, or it can be coupled with other noncognate G proteins that modify the drug pharmacology against A_2A_R. It is worth noting that in ref,^58^ the calculated free energy barrier for the apo-form separating the minima in the “pseudo-active” and inactive states was calculated to be ∼15 kcal/mol for the TM6 rotation and ∼7 kcal/mol for the TM6 translation. In the agonist NECA-bound A_2A_R, the corresponding calculated free energy barriers separating active from inactive region were, correspondingly, ∼13 and ∼12 kcal/mol, with convergence simulation time at 2.4 μs for the apo-A_2A_R, and 3.5 μs for the NECA-bound A_2A_R.

**Coupling of A**_**2A**_ **R with Different Transducer Proteins**

The promiscuous coupling of A_2A_R to Go and cognate Gs protein and the involved conformational selection was studied by Prosser, Sljoka, Picard, and collaborators in 2024 ^167^ using ^19^F solution NMR spectroscopy and appropriately labeled receptors at the cytoplasmic TM6 or TM7 in POPC/POPG nanodiscs. In combination with ^19^F solution NMR functional assays based on GTP hydrolysis and BRET-assay with BRET pair between Gα and Gβγ were applied for testing the G protein selectivity. Additionally, MD simulations were applied for the investigation of the allosteric changes in the activation motifs network (Figure 1), and Monte Carlo (MC) simulations were applied to capture the TM6 or TM7 jumps during the G protein activation.^167^ The study detected two different TM6 major active conformations, **A** ^**TM6**^, **I2** ^**TM6**^, and the minor conformation **I1** ^**TM6**^ (Figures 3 and 4).

It was shown that conformations **A** ^**TM6**^ or **I2** ^**TM6**^ that favor binding with full or partial agonists were coupled preferably to Gs or Go protein, respectively, and are characterized by different allosteric interactions between the OBS and cytoplasmic region where G protein binds. Thus, the MD simulations showed^167^ that the χ_1_ dihedral angle of F286^7.51^ has an inactive-like conformation in the agonist–A_2A_R–Go complex and an active-like conformation in the agonist–A_2A_R–Gs complex. In ref^167^, it was observed that the addition of heterotrimer Gs^GDP^ to the NECA–A_2A_R complex increases the population of the active conformations. However, removal of GDP in the Gs complex stabilizes both **A** ^**TM6**^ and **I2** ^**TM6**^ conformations, while a minor population of **I2** ^**TM6**^ was also observed. A similar population shift was observed toward the active ensemble for A_2A_R in complex with Go^GDP^; however, the nucleotide-free Go complex preferentially stabilizes **I2** ^**TM6**^ and reduces **A** ^**TM6**^ population, indicative of greater conformational heterogeneity and/or dynamics compared to the NECA–A_2A_R–Gs^empty^ complex. Therefore, it seems that A_2A_R facilitates selective coupling with Gs, or noncognate Go has two different fully activated conformational states with larger or smaller volumes of cytoplasmic cavity, respectively, as shown with MD simulations.^167^ These findings warrant further exploration for their significance at the physiological level.

##### 4.1.1.2 Kinetic Measurements

**Exchange between Inactive Conformations and Inactive to Activation Conformations of A**_**2A**_ **R in Micelles and Lipid Bilayers**

The inactive states **S1** ^**ΤM6**^ and **S2** ^**ΤM6**^ in POPC/POPG nanodiscs^153^ are exchanged at a slower rate, i.e., in the ∼2 ms time scale, compared to LMNG micelles.^55^ For the quantification of intramolecular conformational dynamics, Borshchevskiy, Hendrix, and collaborators applied in 2021,^182^ smFRET in A_2A_R reconstituted in POPC/POPG nanodiscs with two dyes linked to the cytoplasmic ends of the TM6 (L225C^6.27^) and H8 (Q310C^8.65^). This study also showed that the inactive conformations are exchanged in low-ms time scale (Figure 3) in agreement with the ^19^F solution NMR spectroscopy of A_2A_R in lipid nanodiscs.^153^

The exchange between inactive and active states of A_2A_R occurs in a low-ms time scale according to ^19^F solution NMR spectroscopy in micelles.^68,153^ The exchange between inactive and active states of A_2A_R also occurs on low-ms time scale (e.g., ∼3 ms) in both apo-state and antagonist-bound A_2A_R in POPC/POPG nanodiscs according to the smFRET data.^182^

Shimada and collaborators in 2020 applied [^13^C,^1^H] solution NMR using the signal intensity changes of ^13^C-labeled Met at cytoplasmic TM6 of A_2A_R in the presence of a partial agonist reconstituted in MNG-3/CHS micelles. The study showed^163^ that there is an exchange between the inactive and active states that occurs at a rate slower than ∼20 ms. Also, the research revealed that the active state dictating receptor’s efficacy includes multiple species (at least two) in equilibria, having different conformations of the NPxxY motif, that are at a faster rate of exchange than the 20 ms scale.^163^

**Exchange between Activation Conformations of A**_**2A**_ **R in Micelles**

smFRET experiments for A_2A_R in MNG-3/CHS micelles measure that the exchange between **I2** ^**ΤM6**^ and **A** ^**ΤM6**^ conformations of A_2A_R in the apo- and partial agonist- or full agonist-bound states in MNG-3/CHS micelles occurs at ≥3 ms.^154^ In ref 154, the fluorescence correlation spectroscopy (FCS), which can follow dynamic quenching due to photoinduced electron transfer (PET), showed that the time scale of the cytosolic TM6 rearrangements ranges from very fast 150 ns to 300 μs motions for the apo-A_2A_R and antagonist-bound A_2A_R. In ref 182, the smFRET of A_2A_R in MNG-3/CHS micelles showed that conformations **I2** ^**ΤM6**^ and **A** ^**ΤM6**^ were in exchange on the sub-ms time scale (300–500 μs) in the agonist-bound A_2A_R state, which has enhanced flexibility for TM6.

#### 4.1.2 Conformations of Cytoplasmic TM7

##### 4.1.2.1 Exploration of the Conformational Equilibrium: Characterization of the Conformers

**a. Studies in Micelles**

Solution ^19^F NMR spectroscopy was applied to labeled A_2A_R at A289^7.54^C in the cytoplasmic TM7 reconstituted in DDM/CHS micelles by Wüthrich and collaborators in 2018.^183^ In ref 183, it was also studied the TM6 (L225^6.27^C)-labeled A_2A_R. Since the TM7 inward reorientation during activation lowers A289^7.54^C solvent exposure, it causes downfield chemical shifts compared to the inactive state and the appearance of conformations **A** ^**TM7**^ and **I2** ^**TM7**^ in the active region of the relevant conformational space. In ref 183, it was reported that compared to the TM6 (L225^6.27^C)-labeled A_2A_R in DDM/CHS micelles in which the conformations **S1** ^**TM6**^, **S2** ^**TM6**^, **A** ^**TM6**^, and **I2** ^**TM6**^ were observed, in the TM7 (A289^7.54^C)-labeled A_2A_R, the conformations **S1** ^**TM7**^, **S2** ^**TM7**^, **A** ^**TM7**^, and **I2** ^**TM7**^ were seen. It was discussed that “**S1**”, “**S2**”, and “**I2**” conformations were similar between TM6- and TM7-labeled samples in DDM/CHS micelles.^183^ In comparison, the solution ^19^F NMR of labeled A_2A_R at the cytoplasmic TM6 in MNG-3/CHS,^68^ LMNG/CHS micelles^55^ LMNG micelles,^80^ and POPC/POPG nanodiscs^153^ showed the presence of conformations **S1** ^**TM6**^, **S2** ^**TM6**^, **A** ^**TM6**^, and **I2** ^**TM6**^, as also reported by Prosser and collaborators in 2017,^165^ using ^19^F NMR in MNG-3/CHS micelles. In ref 183, the EXSY (exchange spectroscopy) 2D and STD (saturation-transfer difference) ^19^F solution NMR experiments of A289^7.54^C-labeled A_2A_R with NECA in DDM/CHS micelles showed a slow exchange between conformations **A** ^**TM7**^ and **I2** ^**TM7**^.^183^ This suggests that conformations **A** ^**TM7**^ and **I2** ^**TM7**^ differ by a high-energy barrier that needs major structural rearrangements of the protein backbone. It was suggested that **A** ^**TM7**^ and **I2** ^**TM7**^ conformations have not been seen in reported crystal structures of A_2A_R. This contrasts with the smFRET study of A_2A_R labeled at cytoplasmic TM4 and TM6 in MNG-3/CHS micelles, which suggested that **A** ^**TM6**^ conformation is a fully activated-like conformation that corresponds to the conformation in experimental structures with PDB IDs 5G53 ^56^ and 6GDG.^85^ While it was suggested by the ^19^F solution NMR and smFRET studies^68154^ that a partial agonist stabilizes the distinct **I2** ^**TM6**^ conformation and a full agonist stabilizes the **A** ^**TM6**^ conformation, it was shown that **I2** ^**TM6**^ and **A** ^**TM6**^ conformations have different PIF conformations according to the [^15^N,^1^H] solution NMR study in LMNG/CHS micelles.^155^ This contrasts with the ^19^F solution NMR spectroscopy of the cytoplasmic TM7-labeled A_2A_R in DDM/CHS micelles,^183^ according to which the addition of a partial agonist showed no effect on the A_2A_R conformation.

In ref 184, it was shown by ^19^F NMR labeled A_2A_R at A289^7.54^C in DDM/CHS that the addition of antagonist ZM241385 reduces the population of **I2** ^**TM7**^ by 13%; the addition of NAM amiloride, which binds the sodium-binding pocket, increased the population of **I2** ^**TM7**^ by 5%; the addition of NAM Fg754 increased the population by 8% increased the population of **I2** ^**TM7**^.^184^ Notably, the line width of state **I2** ^**TM7**^ was reduced by 30 Hz in the presence of ZM241385 but increased by 70 Hz in the presence of hexamethylene amiloride (HMA) or Fg754, pointing to different conformational dynamics of the receptor bound with an orthosteric antagonist versus a NAM.^69^ Therefore, the ^19^F NMR analysis suggests that Fg754 may induce a specific conformational ensemble of A_2A_R that is different from the effect of HMA or ZM241385. It warrants further investigation as to how different binding poses of Fg754 and HMA could give rise to their differential effects in modulating the receptor conformational equilibrium.

**b. Studies in Lipid Bilayers**

**A**_**2A**_ **R Conformation**

Prosser, Sljoka, Picard, and collaborators in 2024,^167^ reported that the solution ^19^F NMR spectra of TM7-labeled A_2A_R in POPC/POPG nanodiscs showed the presence of conformations **S1** ^**TM7**^ and **I1** ^**TM7**^, **I2** ^**TM7**^, **A** ^**TM7**^, with an equal population between the inactive and active conformation.^167^

Eddy and collaborators observed in 2024 ^157^ with solution ^19^F NMR of the A289^7.54^C-labeled A_2A_R in the presence of a partial agonist (regadenoson or LUF5834) in POPC/POPS nanodiscs at 280 K, the presence of a previously unseen active intermediate **I** ^**ΤM7**^ is in a lower population compared to conformations **I1** ^**TM7**^, **I2** ^**TM7**^, and **A** ^**TM7**^. The **I** ^**ΤM7**^ conformation was observed in the spectra of the A_2A_R–partial agonist complex at all recorded temperatures, 280, 298, 310 K (7, 25, 37 °C). The **I** ^**ΤM7**^ conformation was not previously found in the spectra of apo-A_2A_R, agonist-bound, or antagonist-bound A_2A_R. This distinct conformation in the active region might either bind and activate Gs or may facilitate interactions between the A_2A_R and regulatory proteins such as GRKs and/or β-arrs, which are necessary for internalization of the receptor and other signaling pathways.

Eddy and collaborators published in 2025 ^185^ a ^19^F NMR magic angle spinning solid-state NMR (MAS ssNMR) study in A289^7.54^C-labeled A_2A_R in POPC/POPS liposomes with the same ratio as in their previous lipid nanodiscs studies.^186^ Using this liposome formulation, the presence of the inactive conformations **S1** ^**ΤM6**^ and **S2** ^**ΤM6**^ and additional states in the inactive region was observed. The conformation **I2** ^**TM7**^ was not observed, while conformations **A1** ^**ΤM7**^ and **I1** ^**ΤM7**^ were the most populated.

Lamichhane, Eddy, and collaborators in 2023 ^187^ studied with SMF/TIRF the conformational equilibrium of A_2A_R labeled at the cytoplasmic end of TM7 in POPC/POPS/POPE (1-palmitoyl-2-oleoyl-*sn*-glycero-3-phosphoethanolamine) nanodiscs. Three distinct mutant receptors were also produced with the I92^3.40^N or D52^2.50^N mutations near the conserved “toggle switch” W246^6.48^, increasing or decreasing A_2A_R basal activity, respectively, or with the cytoplasmic R291^7.56^Q increasing basal activity. The I92^3.40^N and R291^7.56^Q are called constitutively activating mutations (CAM) since they can increase receptor basal activity. Similar changes to signaling have also been caused by mutations in the same locations in several other class A GPCRs. The mechanism by which the signaling activity of A_2A_R D52^2.50^N is reduced has not been decided, but previous crystal structure determination of the full agonist UK432097–D52^2.50^N A_2A_R complex (PDB ID 5WF5 ^188^) in LMNG/CHS micelles by White, Stevens, and collaborators in 2018 ^188^ showed subtle changes in the backbone orientation close to the conserved NPxxY motif. This is due to the destabilization of the sodium-binding motif by the mutation D52^2.50^N, causing a reduction in G protein signaling. As previously mentioned, [^15^N,^1^H] solution NMR experiments by Wütrich and collaborators in 2018 ^188^ showed that this mutation altered the conformational dynamics of the cytoplasmic region, which are related to its function. In the SMF/TIRF studies, all 4 receptors, A_2A_R, D52^2.50^N A_2A_R, I92^3.40^N A_2A_R, and R291^7.56^Q A_2A_R, showed three conformations: one inactive conformation and two conformations in the active region for the agonist NECA-bound state, the apo-state, and the antagonist ZM241385-bound state. In the apo-A_2A_R and presence of antagonist ZM241385 were observed a 63% inactive state (that might include conformations **S1** ^**TM7**^, **S2** ^**TM7**^ seen by solution ^19^F NMR studies) and 37% of active conformation that might correspond to **I2** ^**TM7**^ seen in the ^19^F NMR studies. For agonist-bound A_2A_R, the inactive conformation was reduced (49%), the **I2** ^**TM7**^ conformation remained in the same population (37%), and a new active conformation in 14% population appeared (possibly conformation **A** ^**TM7**^ observed with ^19^F NMR), in agreement with previous results.^189^ Compared to A_2A_R, in all samples with A_2A_R D52^2.50^N, the population of the inactive state was increased and the population of the active state was reduced in agreement with basal activity; the addition of an agonist did not increase the population of the conformations in the active region, indicating that the mutation eliminated the sensitivity of the receptor to the efficacy of bound ligands. Compared to A_2A_R, in the samples with CAM (apo-receptors, complexes with antagonist and with agonist), a reduction in the population of the inactive state, an increase in the population of **I2** ^**TM7**^, and a significant increase in the population of state **A** ^**TM7**^ were measured. Activation kinetics show that CAMs increase the frequency of transitions to the intermediate state through which transition to the active state is achieved.

The addition of mini-Gs increased the population of **A** ^**TM7**^ conformation, postulating its identity as a fully activated-like conformation: for apo-A_2A_R and antagonist-bound A_2A_R, the population was <1% before addition and 8% after addition of mini-Gs, while for the agonist NECA-bound A_2A_R, the population increased from 14% to 21% after addition of mini-Gs. In all samples of A_2A_R, upon mini-Gs addition, the population of the inactive state decreased, while the population of **I2** ^**TM7**^ was almost unaffected. The addition of mini-Gs in receptors with CAMs slightly affects the relative populations, suggesting that it does not further shift the equilibrium of the conformations, likely because it is notably distinct from the G protein contact region.

Wüthrich and collaborators in 2024 ^190^ compared previous solution ^19^F NMR results with those obtained with A_2A_R reconstituted in LMNG/CHS micelles or POPC/POPS (1-palmitoyl-2-oleoyl-*sn*-glycero-3-phosphoserine) nanodiscs, finding no obvious differences.

**Coupling of A**_**2A**_ **R with Different Transducer Proteins**

Prosser, Sljoka, Picard, and collaborators explored^167^ with ^19^F solution NMR of A289^7.54^C-labeled A_2A_R in POPC/POPG nanodiscs, the coupling of A_2A_R with full agonist and with cognate Gs and noncognate Go proteins, and the resulting changes upon removal of GDP. When the full agonist NECA was added to the apo-receptor sample, the population of the active state conformations **I1** ^**TM7**^, **I2** ^**TM7**^, and **A** ^**TM7**^ increased while conformation **S1** ^**TM7**^ was also observed. Further, upon addition of G^GDP^ protein to A_2A_R, the corresponding complexes from conformations **I1** ^**TM7**^, **I2** ^**TM7**^, and **A** ^**TM7**^were formed; **S1** ^**TM7**^ disappeared upon addition of Gs^GDP^ but was only reduced (to ∼37%) with Go^GDP^. In the case of Gs^GDP^ protein, the **I2** ^**TM7**^ conformation was mostly favored, while in the case of Go^GDP^, all active states were equally populated. Removal of GDP shifts the equilibrium toward **A** ^**TM7**^ conformation in both Gs^empty^ and Go^empty^ complexes, although in the case of the Go^empty^ complex, a significant percentage of **I2** ^**TM7**^ and **I1** ^**TM7**^ conformational states remained, indicating the lower level of activation efficiency by the Go protein. While the higher-efficacy active conformations **A** ^**TM6**^ and **A** ^**TM7**^ are more populated after GDP removal, **I1** ^**TM7**^, which was more stable in the presence of GDP (than in the absence of the nucleotide), presumably represents an activation intermediate conformation. The populations of the TM6 and TM7 activation states may be correlated, albeit they are not identical.

##### 4.1.2.2 Kinetic measurements

**a. Studies in Lipid Bilayers**

**Exchange between Inactive and Activation Conformations of A**_**2A**_ **R**

It is expected that conformational states have a slower exchange rate in phospholipid bilayers compared to detergent micelles. It was shown with ^19^F solution NMR spectroscopy by Prosser, Sljoka, Picard, and collaborators in 2024 ^167^ that the **S1** ^**TM7**^ and **I1** ^**TM7**^ conformations in lipid nanodiscs showed exchange on the sub-ms time scale in the A_2A_R–Go complex, suggesting the possible role of this dynamic exchange in activation of A_2A_R. In comparison, the results in micelles showed a low-ms time scale for the exchange of cytoplasmic TM6 between inactive and active states of A_2A_R.

**Detection of Very Slow and Very Fast Motions in A**_**2A**_ **R**

This was accomplished with SMF/TIRF of A_2A_R linked with a fluorophore Cy3 covalently attached at cytoplasmic A289^7.54^C reconstituted in POPC/POPS/POPE-N-(cap biotinyl) (biotinyl CAP PE) nanodiscs by Lamichhane, Eddy, and collaborators in 2022 ^187^ detecting exchange between conformational states on time scales even longer than 100 ms. These SMF/TIRF experiments showed in the agonist-bound A_2A_R state faster dynamics (390 ± 80 μs).^189^ Interestingly, direct exchange between the inactive and the active TM7 conformations was not observed.^189^

Lamichhane, Eddy, and collaborators in 2023,^187^ performed an additional SMF/TIRF study of A_2A_R, labeled at the cytoplasmic end of TM7 in POPC/POPS/POPE nanodiscs. It was shown that in A_2A_R or A_2A_R D52^2.50^N, the occupancy of the inactive state was longer (∼2.9 s) compared to the time spent in the active state in samples of the apo-A_2A_R, antagonist-bound A_2A_R, agonist-bound A_2A_R, and A_2A_R D52^2.50^N. In A_2A_Rs with a CAM, it was measured in the inactive state a shorter occupancy time (∼1.5 s) compared to A_2A_R and A_2A_R D52^2.50^N. The addition of mini-Gs reduced the occupancy times in the inactive state of A_2A_R, especially for agonist-bound A_2A_R (∼1.8 s) and did not affect any of the A_2A_R D52^2.50^N or A_2A_R R291^7.56^Q, or A_2A_R I92^3.40^N.

### 4.2 β_2_ Adrenergic Receptor

Ligand or transducer protein binding to β_2A_R can induce several conformational states related to the receptor’s intracellular, transmembrane, and extracellular domains.

#### 4.2.1 Conformations of the Extracellular and TM Regions

##### 4.2.1.1 Exploration of the Conformational Equilibrium: Characterization of the Conformers

**a. Studies in Micelles**

The binding of an orthosteric ligand in the extracellular region of the β_2_AR causes an allosteric conformational alteration in the intracellular part of the receptor. This effect was studied by Kobilka and collaborators in 2010,^191^ using [^13^C,^1^H] solution NMR spectroscopy in ε-N­[^13^CH_3_] (^13^C-dimethyllysine-labeled) labeled β_2_AR reconstituted in DDM micelles. The drugs tested had different efficacy (agonist, neutral antagonist, or inverse agonist) toward G protein activation. The K305^7.32^-D192^EL2^ salt bridge connecting ECL2 to ECL3 and TM7 is a characteristic feature of the extracellular surface of β_2_AR, as is shown, for example, in the X-ray structure of the unliganded β_2_AR in the inactive conformation reported in ref 191 (PDB ID 2R4S^191^). Changes in the NMR spectrum as regards the residues of K305^7.32^-D192^ECL2^ salt bridge indicate conformational changes in ECL2 as revealed by the ^13^C-dimethyllysine signals of K305^7.32^. The level of the inspected perturbation in ECL2 conformation depends on the ligand’s efficacy, with antagonists causing no change, in contrast to full agonists. The NMR results coupled to MD simulations suggested^191^ that drugs binding to the OBS in the extracellular region can alter receptor function since perturbation in EL2 propagates conformational changes in cytosolic TM6 and TM7 that couple to Gs protein. Thus, upon binding in the OBS of β_2_AR by full agonist formoterol, the agonist’s β-hydroxyl group binds to N293^6.55^, causing the inward motion of the extracellular end of TM7, which is followed by the outward motion of the cytoplasmic end of TM6 toward TM5. Three different conformations were detected in the extracellular region of β_2_AR: one for the apo-β_2_AR or the neutral antagonist (alprenolol)-bound state, one for the inverse agonist (carazolol)-bound state, and one for the full agonist (formoterol)-bound state, resulting in different functional responses. It seems that both the apo-state and antagonist alprenolol-bound β_2_AR can couple Gs, which agrees with the basal activity of the receptor and neutral antagonist alprenolol’s efficacy,^192^ that full agonists, e.g., formoterol, produce the strongest coupling, while the inverse agonist carazolol did not allow coupling of the receptor with Gs protein. Kobilka, Lefkowitz, and collaborators reported in 2019 the X-ray structure of the fully active state of β_2A_R bound to the full agonist BI-167107, Nb6B9 as Gs protein, mimetic and the PAM Cmpd-6FA (PDB ID 6N48 ^193^) showing that PAM binding in the cytoplasmic region at the lipid interface caused allosterically the enhancement of orthosteric agonist binding BI-167107 and the stabilization of the fully active conformation of the β_2_AR. Indeed, the GaMD simulations by Miao and collaborators in 2023 ^172^ showed that Cmpd-6FA binding causes a rigidity of residues in the OBS included by ECL1 and extracellular TM7 end and the intracellular TM3 as well as ICL1 and ICL2, as also shown in the structural study in ref 193.

Shimada and collaborators in 2012 ^194^ also applied solution [^13^C,^1^H] NMR spectroscopy with ε-N­[^13^CH_3_] labeled β_2_AR in the extracellular and TM regions, and the receptor was reconstituted in DDM micelles. Kobilka, Jin, and collaborators, in 2013 ^91^ used the same solution NMR spectroscopy method with ε-N­[^13^CH_3_]-labeled β_2_AR reconstituted in DDM/CHS micelles in combination with MD simulations. The receptor was labeled at OBS, TM, and the cytoplasmic region. Indeed, the ^13^C chemical shift changes of Met82^2.53^, Met215^5.54^, and Met279^6.41^ were examined, showing conformational changes in different regions of the receptor. The Met82^2.53^ site is sensitive to the chemical environment surrounding the ligand–OBS complex, since it lies in TM2 below and close to OBS, i.e., at a 4–5 Å distance close to many amino acids that interact with both agonists and antagonists. Met215^5.54^ and Met279^6.41^ sites are sensitive as regards the conformational changes needed for the receptor’s activation, located in the TM region between the OBS and cytoplasmic ends of TM5 and TM6, respectively, that undergo significant structural modifications to enable binding to Gs. Based on the resonances from Met82^2.53^, it was identified that in the apo-form or when the β_2_AR binds the neutral antagonist alprenolol or the inverse agonist carazolol, two major inactive conformations were observed in equilibration with a conformation in the active region in a minor population; the conformations differ mainly in the conformation close to the OBS. This equilibrium may correlate with the basal activity even in the presence of an inverse antagonist.^192^ It was shown^194^ in the DDM micelles, the full agonist formoterol or carazolol binding forms an intermediate active conformation, and the partial agonist tulobuterol or clenbuterol binding causes the formation of a conformation with a profile between an inactive and intermediate active conformation. The relative population of these inactive and active intermediates reflects the relative efficacy of tulobuterol and clenbuterol. It seems by NMR that the M82^2.53^ chemical shifts show a ligand efficacy-dependent conformational equilibrium, which must be accompanied by significant conformational changes on cytosolic TM5 and TM6. A distinct weakly populated active state was observed in DDM/CHS micelles^91^ in the presence of agonists, which is stabilized through binding of a Gs protein mimetic (Nb80).

#### 4.2.2 Conformations of the Cytoplasmic Region

##### 4.2.2.1 Exploration of the Conformational Equilibrium: Characterization of the Conformers

**a. Studies in Micelles**

**β**_**2**_ **AR Conformation**

Kobilka and collaborators studied in 2006 ^195^ with fluorescence microscopy, a modified β_2_AR receptor reconstituted in DM/CHS micelles with fluorescent reporter groups attached at the cytoplasmic TM3 and TM6 ends. The study aimed to monitor how the “ionic lock” R^3.50^-E^6.30^ interaction is modified by the addition of ligands in the apo-β_2_AR sample. In the inactive conformation of apo-β_2_AR, the distance between Cα carbons of A271^6.33^ and I135^3.54^ is ∼11 Å. A mutant A271^6.33^C, I135^3.54^W-β_2_AR was labeled at A271^6.33^C with a bimane dye, which is a fluorophore sensitive to its environment. When the fluorescent probe groups bimane and tryptophan were at 5–15 Å, quenching of bimane fluorescence by tryptophan reported an interaction. It was observed that full agonists and partial agonists disrupt the “ionic lock” interaction.^195^

Wüthrich, Stevens, and collaborators studied in 2012 ^196^ with ^19^F solution NMR, the β_2_AR in DDM/CHS micelles. The receptor was labeled at the cytoplasmic ends of TM6 (C265^6.27^) and TM7 (C327^7.54^), which showed large conformational changes during activation, and at the C-end of non-transmembrane H8 (C341). Upon addition of Gs-biased agonists (e.g., isoproterenol) or β-arr1-biased ligands (agonist isoetharine and β-blocker carvedilol), these solution ^19^F NMR studies of β_2_AR identified distinct active-like conformations for coupling with G protein or β-arr protein, respectively.^196^ The Gs-biased agonists shifted the equilibrium toward the Gs-specific active state by perturbing the conformation of cytosolic TM6 compared to the β-arr1-biased agonists (e.g., isoetharine) that mainly impact the conformation of the TM7 cytoplasmic end. Previous research (see discussion in the Signaling complexes of class A GPCRs section, part B) with a variety of class A GPCRs indicates that TM7 mediates receptor coupling to β-arr and signaling bias. Binding of both a Gs-biased or a balanced agonist (formoterol) to β_2_AR results in an equally populated equilibrium of the active and inactive cytoplasmic TM6 conformation. However, the binding of a β-arr-biased agonist (isoetharine) produced only the active-like TM7 cytoplasmic conformation, whereas in the presence of the balanced agonist formoterol, the inactive state was still significantly populated by ∼35%.^196^

Using trimethylsilyl (TMS) as a reporter group attached to the cytoplasmic TM6 (C265^6.27^) or TM7 (C327^7.54^) of β_2_AR in DDM/CHS micelles, Liu and collaborators in 2019 examined the effect of various ligands on TM6 or TM7 conformation and produced results in agreement with ref 196. The antagonists alprenolol and carazolol produced marginal changes in the ^1^H NMR signals of these two constructs, suggesting that they keep β_2_AR in a predominant inactive conformation, as in the apo-form. The β-arr-biased ligand carvedilol produced an equilibrium consisting mainly of the C327^7.54^ active state but kept C265^6.27^ mainly in the inactive state. The balanced agonist formoterol produced the greatest conversion toward the active state of β_2_AR, as was observed by the ^1^H NMR signals of TM6 (C265^6.27^) or TM7 (C327^7.54^).

In a subsequent study, Wüthrich and collaborators in 2016,^197^ reported results from the application of ^19^F solution NMR β_2_AR and T4L-β_2_AR labeled at cytoplasmic TM6 and TM7 (similarly to the study in ref 196) in DDM/CHS micelles. It was shown^197^ that compared to β_2_AR, the effect of T4L linked to IL3 of β_2_AR (residues 231 to 262 in ICL3, connecting TM5 and TM6, were replaced by residues 2 to 164 of the T4L protein), is to cause the population of only active TM6 conformations independent of the efficacy of bound ligands. This contrasted with TM7 conformations, suggested to be involved with engagement to β-arr, where the population of both active and inactive conformations was seen, as also observed in ref 196 with β_2_AR. This agrees with the relatively high level of basal signaling activity of β_2_AR. Millar, Wüthrich, Stevens, and collaborators reported in 2015 ^198^ SMF/TIRF microscopy results in β_2_AR reconstituted in POPC/POPS/biotinyl CAP PE nanodiscs labeled with the cyanine Cy3 fluorescence probe at C265^6.27^. Millar and collaborators in the study of 2020,^199^ used β_2_AR labeled at C327^7.54^ with fluorescent probe Cy3, located near the cytoplasmic end of TM7, and reconstituted in a DDM/CHS environment using SMF microscopy. In both publications,^198,199^ it was shown that apo-β_2_AR equilibrates between an active and an inactive state, TM6 ^198^ or TM7 ^199^ conformations. In ref 199, the effect of a balanced agonist (e.g., formoterol) and β-arr-biased agonist (isoetharine) on the conformational dynamics of TM7 was compared. It was shown that both ligands act not through destabilization of the inactive state but by kinetic stabilization of the active state and extending their residence time inside the receptor.

Kobilka and collaborators reported in 2013 ^91^ or Kobilka, Jin, and collaborators reported in 2020,^200^ solution NMR studies with β_2_AR N­[^13^CH_3_]-labeled in the OBS/TM/cytoplasmic region or the cytoplasmic region, respectively, with the receptor reconstituted in DDM/CHS micelles. Compared to the [^13^C,^1^H] solution NMR spectroscopy of ^13^CH_3‑_labeled ε-Met-β_2_AR in DDM micelles,^194^ in the DDM/CHS micelles study^91^ in addition to ^13^C chemical shift changes of Met82^2.53^, Met215^5.54^, and Met279^6.41^ in the extracellular and TM domains, the L272^6.34^ M in the cytoplasmic TM6 was also explored. Based on the cytoplasmic TM6 conformations, the unliganded state or inverse agonist carazolol-bound state showed the presence of one inactive conformation (possibly **S1** ^**ΤM6**^). In the presence of the high-affinity, high-efficacy agonist BI-167107 without Nb80, the NMR experiments revealed for the cytosolic TM region of β_2_AR the presence of the active conformation **I2** ^**ΤM6**^ with a reduction of the population (destabilization) of the inactive state. Addition of BI-167107 to apo-β_2_AR and Nb80 showed in predominance, the signal of the active conformation **A** ^**ΤM6**^, which thus corresponds to a fully activated-like conformation, with a different ^13^C chemical shift from **I2** ^**ΤM6**^.^91^ Thus, the active conformations **A** ^**ΤM6**^ and **I2** ^**ΤM6**^ were differentiated based on these solution ^13^C NMR data in the DDM/CHS micelles. It was suggested that the **A** ^**ΤM6**^ conformation corresponds to the conformation that β_2_AR has in the X-ray structure of the BI-167107−β_2_AR–Gs complex (PDB ID 3P0G^201^), and the **I2** ^**TM6**^ conformation corresponds to the conformation that β_2_AR has in the X-ray structure of the full agonist FAUC50−β_2_AR complex (PDB ID 3PDS^160^). The coupling of β_2_AR with Gαs has been explored with convenient MD simulations and GαMD by Murarka and collaborators in 2024 ^202^ using the full agonist BI-167107−β_2_AR complex or the BI-167107−β_2_AR–Gαs complex.^202^

Prosser and collaborators reported in 2013 ^203^ a solution ^19^F NMR study in C265^6.27^-labeled β_2_AR in MNG-3 micelles. Kobilka and collaborators reported in 2015 a solution ^19^F solution NMR and DEER spectroscopy in β_2_AR, reconstituted in mixed MNG-3/CHS micelles.^69^ The receptor was labeled, correspondingly, at the cytoplasmic TM6 (C265^6.27^C, L266^6.28^C), TM4 (N148^4.40^C) with nitroxide probes. The ^19^F solution NMR of labeled β_2_AR at the cytoplasmic half of TM6 reconstituted in mixed MNG-3/CHS micelles^69^ and in MNG-3 micelles,^203^ or the SMF/TIRF microscopy of β_2_AR reconstituted in lipid nanodiscs^198^ has revealed for the receptor in the unliganded state the presence of two inactive conformations, **S1** ^**ΤM6**^ and **S2** ^**ΤM6**^, that undergo rapid transitions. Conformations **S1** ^**ΤM6**^ and **I2** ^**ΤM6**^ have a stabilized “ionic lock” between TM3 and TM6 and a broken “ionic lock” conformation, respectively, demonstrated by measurements of distances between the site-specific spin labeling at the intracellular ends of TM4 and TM6 by DEER spectroscopy.^69^ The broken “ionic lock” conformation **S1** ^**ΤM6**^ was the predominant inactive conformation.^69^ This agrees with predictions from MD simulations by Shaw and collaborators reported in 2009,^204^ which identified the presence of this inactive conformation in unliganded β_2_AR.^204^ These findings, which are in agreement with the fluorescence microscopy results in DM/CHS micelles published by Kobilka and collaborators in 2006,^195^ demonstrated that complete activation of the β_2_AR requires, but is not dependent upon, the disruption of this important molecular switch motif. Upon agonist addition, the conformation in the active region **I2** ^**TM6**^ was observed.^198,203^ According to the DEER spectroscopy results,^69^ **I2** ^**TM6**^ conformation represents a transiently populated, active conformation that, upon agonist binding, helps the change from inactive to active conformation by decreasing the activation energy of the TM6 pivoting motion. Müller, Kobilka, and collaborators applied atomic force microscopy (AFM)-based SMF microscopy in β_2_AR reconstituted in liposomes composed by DOPC and cholesterol and reported in 2012 ^205^ that the free energy barrier required for the motions of the structural segments of unliganded β_2_AR, as regards an energy stabilized conformation, is ∼52–72 kJ/mol (∼12–17 kcal/mol). Thus, β_2_AR samples additional conformations, and this agrees with β_2_AR’s basal activity and the binding by many ligands.

The inactive conformations **S1** ^**ΤM6**^ and **S2** ^**ΤM6**^ predominate over the active conformation **I2** ^**TM6**^ in the presence of an inverse agonist, as was observed with ^19^F NMR in MNG-3 micelles^203^ or ^19^F NMR and DEER spectroscopy in MNG-3/CHS micelles,^69^ or in the presence of a neutral antagonist, as observed with ^13^C NMR in the DDM/CHS micelles study.^91^ The inactive conformations **S1** ^**ΤM6**^, **S2** ^**ΤM6**^ predominate (with broken “ionic” conformation **S2** ^**TM6**^ exhibiting the highest population)^206^ even in the presence of the low-affinity, full agonist isoproterenol (15–20% **S2** ^**TM6**^) as observed by ^19^F NMR in MNG-3 micelles^69^ or by ^19^F NMR and DEER spectroscopy in MNG-3/CHS micelles.^69^ The basal activity of β_2_AR can be attributed to the presence of a significant population of active-like states with a calculated population of 38%, reported by Delemotte and collaborators in 2023 ^207^ using enhanced sampling MD simulations based on the accelerated weight histogram (AWH) method, in agreement with the 40% population observed in the ^19^F NMR study in MNG-3 micelles, even in the presence of an inverse agonist.^203^

Only in the presence of the ultrahigh-affinity full agonist BI-167107, the population of the two inactive conformations **S1** ^**ΤM6**^ and **S2** ^**ΤM6**^ in the equilibrated mixture was reduced to 40–50% as revealed by DEER spectroscopy of β_2_AR in MNG-3/CHS micelles.^69^ The [^1^H,^13^C] solution NMR spectra of the β_2_AR reconstituted in DDM/CHS micelles showed^200^ that upon addition of the ultrahigh-affinity full agonist BI-167107, conformational changes in cytoplasmic TM4, TM5-TM7 were observed that form the conformations in the active region,^200^ including the outward movement of cytoplasmic TM6 toward TM5 and the inward movement of cytoplasmic TM7, which is needed for the insertion of the Cα5 helix of Gαs in the presence of Gαs. These conformational changes were also observed by fluorescence spectroscopy in DDM/CHS micelles as reported by Kobilka and collaborators in 2004.^208^

Shimada and collaborators studied in 2020 ^162^ with [^1^H,^15^N] solution NMR of leucine backbone amide groups the β_2_AR reconstituted in LMNG micelles. The results suggested that the apo-β_2_AR without thermostabilizing mutations populates the inactive **S1** ^**ΤM6**^ conformation and active conformation **I2** ^**TM6**^. The paramagnetic relaxation enhancement (PRE) experiments showed^162^ that **I2** ^**TM6**^ and **S1** ^**ΤM6**^ conformational states have similar structure in the PIF motif and TM6 cytosolic conformation, revealing the weak coupling between the OBS and cytoplasmic region in conformation **I2** ^**TM6**^, as has also been suggested by MD simulations of β_2_AR by Shaw and collaborators in 2011.^209^ The **I2** ^**TM6**^ conformation likely corresponds to the conformation observed in the X-ray structures of only agonist-bound β_2_AR (e.g., PDB ID 2Y02 ^161^), which is similar to the inactive conformation in the X-ray structures of β_2_AR bound to the inverse agonist ICI 118,551 (PDB ID 3NY8 ^67^) or the antagonist alprenolol (PDB ID 3NYA^67^). The binding of an inverse agonist shifts the equilibrium toward the inactive conformation **S1** ^**ΤM6**^, the binding of a full agonist (formoterol or isoproterenol) shifts the equilibrium toward conformation **A** ^**ΤM6**^, while the presence of a partial agonist causes a submaximal population of the active conformation **I2** ^**TM6**^.^162^ Conformations **I1** ^**ΤM6**^ and **I2** ^**TM6**^ are both in exchange, while, compared to **S1** ^**ΤM6**^ or **I2** ^**TM6**^, conformation **A** ^**TM6**^ exhibits a major TM6 outward pivotal movement associated with a large conformation change in the PIF motif.^162^

**Coupling of β**_**2**_ **AR with Different Transducer Proteins**

Blanchard, Kobilka, and collaborators in 2017 ^97^ published results on the investigation of the movements of the TM6. It was used smFRET spectroscopy of full-length β_2_AR in DDM micelles labeled at the cytoplasmic ends of TM4 (N148C^4.40^) and TM6 (L266C^6.28^) with an optimized Cy3B and Cy7 fluorophore pair (Cy3B* and Cy7*, respectively). The study showed,^97^ as well as the above-mentioned studies (i.e., the ^19^F NMR and DEER spectroscopy in MNG-3/CHS micelles,^69^ or the solution ^13^C NMR spectroscopy in DDM/CHS micelles),^200^ that the fully activated conformation **A** ^**TM6**^ becomes dominant upon addition of a Gs or Gi1 protein or Nb80. The **I2** ^**TM6**^ conformation of agonist-only bound β_2_AR is different from that of **A** ^**TM6**^, since, according to the X-ray or cryo-EM structures, the agonist binding alone cannot stabilize the fully active conformation. While the changes observed in the NMR spectra and MD simulations for the coupling of β_2_AR with Gs or Gi1 proteins were similar, a significant exception was shown in ICL2, which was more flexible in the Gi1-bound conformation and could not form an α-helix that supports a tighter binding in the case of Gs.^200^ This is important since G protein forms critical interactions with ICL2 for receptor activation, and the difference in ICL2 may represent a critical determinant for the selective Gs protein coupling and activation. Recall that β_2_AR is preferentially coupled with Gs over Gi1. A large binding pocket in the intracellular core of GPCR can reflect the ability of the receptor to couple with multiple G proteins. This was shown through comparison of experimental structures with different G proteins (see previous discussion on the conformations of cytoplasmic TM7 of A_2A_R). It can be thought that GPCRs that are primarily coupled to Gs and Gq can also promiscuously couple Gi, as shown by Lambert and collaborators with BRET-based assays in cells in 2019.^210^ Remarkably, in the cryo-EM structure of the complex agonist LM189−β_2_AR–Gi (PDB ID 9BUY^211^), reported by Kobilka, Lerch, Gmeiner, and collaborators in 2024,^211^ showed that the outward movement of cytosolic TM6 is smaller compared with structures of agonist−β_2_AR–Gs complexes. The combination of smFRET and DEER in ref 211 also showed that the Gαi-biased agonist LM189, compared to Gs complexes, adopts a distinct conformation when coupled to Gi (see discussion in the last section of the review).

NMR investigations and MD simulations published by Su, Wand, and collaborators in 2020 ^212^ explored the conformational changes of β_2_AR upon coupling to β-arr1 through monitoring the ^1^H NMR spectra of the 4-(trimethylsilyl)­phenylalanine (TMSF) group in samples of receptor reconstituted in LMNG/CHS micelles. The TMSF group was connected in a β-arr1 via genetically encoded technology. Compared to ^19^F NMR probes, the NMR experiments using this TMSF probe required a substantially smaller amount of protein and accumulation time. Using this trimethylsilyl ^1^H NMR probe in samples of phospho-β_2_AR−β-arr1 complexes, multiple conformational states of the β_2_AR were detected in the signaling complex. The presence of these conformational states seems to be controlled by interactions of β-arr1 with the TM core and the C-terminal tail of the receptor. Some of these conformers may be associated with different receptor states during signaling, which can also contribute to G protein selectivity and ligand efficacy.

**b. Studies in Lipid Bilayers**

**β**_**2**_ **AR Conformation**

GaMD simulations by Tikhonova and collaborators reported in 2013 ^213^ predicted for the unliganded state of β_2_AR the presence of three closed-in cytoplasmic region conformations, which can correspond to the **S1** ^**TM6**^, **S2** ^**TM6**^, and **I2** ^**TM6**^ observed in the NMR studies.^200^ In the presence of a G protein-biased agonist, e.g., fenoterol, an open conformation in the cytoplasmic region was observed, which may correspond to an **A** ^**ΤM6**^-like conformation. In complexes with β-arr biased agonists (e.g., *N*-cyclopentylbutanepherine), the β_2_AR samples are in the inactive conformation **S1** ^**TM6**^ energy basin. In the MD simulations, the β-arr-biased agonists caused an inward, counterclockwise twist of TM7 (P^7.50^ and Y^7.53^ in the N^7.49^P^7.50^xxY^7.53^ motif), which decreases the intracellular cavity opening, favoring β-arr coupling. This was detected in X-ray structures of complexes with arrs, e.g., the crystal structure of RhoR–visual arr1 reported by Zhou, Xu, and collaborators in 2016 ^214^ (PDB ID 5DGY^214^) and the XFEL structure of RhoR bound to β-arr (PDB ID 4ZWJ^215^), which showed that a large binding cavity in the cytosolic region should hinder arr coupling. Comparatively, as was shown in the MD simulations, fenoterol causes the largest TM2-TM7 distance, while the β-arr-biased agonist *N*-cyclopentylbutanephrine causes the smallest distance. Pu and collaborators performed in 2021 ^216^ GaMD simulations using complexes of β_2_AR with different Gs- or β-arr biased agonists, or Plazinski, Plaszinska, and collaborators reported in 2024 ^217^ convenient MD simulations and metadynamics-based binding free energy calculations of β_2_AR with different agonists and CG MD simulations of restrained agonist-induced conformations of the receptor. Similarly, it was shown that the agonist in the extracellular region caused formation in the cytoplasmic region of a binding area of a different size for coupling to Gs or β-arr. Goddard III and collaborators published in 2023 ^218^ metadynamics simulations, exploring details of the mechanism of β_2_AR activations. Some structural changes from structural biology were reported, e.g., it was observed that the ionic lock is not broken by agonist binding to the inactive β_2_AR alone, which prevents the β_2_AR from moving toward the activated conformation. Nevertheless, it was observed that when the inactive Gs (Gs^empty^) is attached to the agonist-bound inactive β_2_AR (which has the ionic lock), Gαs-α5 helix partially inserts into the β_2_AR core, breaking the ionic lock and activating the Gs protein connected to β_2_AR. When the Gαs protein is activated, the GDP binding pocket opens remarkably, allowing the GDP to be released or exchanged. At the same time, when the ionic lock is broken, Gαs-α5 experiences a remarkable expansion in the β_2_AR cytoplasmic region, causing TM6 to displace outward by ∼5 Å from TM3.^218^

**Coupling with Different Transducer Proteins**

Kobilka and collaborators reported in 2019 ^219^ the effect of phospholipid content of the membrane on coupling of β_2_AR with Gs and Gi proteins using fluorescence spectroscopy of β_2_AR labeled at C265^6.27^ in cytoplasmic TM6 with monobromobimane (mB). The (mB−β_2_AR) was reconstituted in DOPS and DOPG nanodiscs. It was found that phospholipids with a negative charge improve β_2_AR binding with Gs while hindering contact with Gi. Furthermore, it was shown that Ca^2+^ and Mg^2+^ cations promote the β_2_AR–Gi interaction in negatively charged lipids, suggesting that the β_2_AR interaction with Gi is modulated by local membrane charge, which is adjusted by intracellular cations.

In the same context as the study of Blachard, Kobilka, and collaborators in 2017, described previously,^97^ MD simulation results were reported by Hildebrand and collaborators in 2014 ^220^ in complexes of β_2_AR with C-terminal peptides of Gα embedded in DMPC bilayers to mimic the interaction between β_2_AR and Gαβγ. The study aimed to explain the coupling selectivity of β_2_AR to Gi vs Gs. Τhe C-terminal peptides of Giα and Gsα form the main interaction of β_2_AR with Gα of Gαβγ. In complexes of β_2_AR with C-terminal peptides of Gα, the thinner C-terminal peptide of Giα stabilizes a receptor conformation not accessible to the bulkier C-terminal peptides of Gsα, which need a bigger TM6 outward tilt for binding. Thus, the C-terminal peptides of Giα and Gsα bind unique cytoplasmic receptor conformations that coexist in the uncomplexed β_2_AR due to the flexibility of TM6.

**c. Studies in Cells**

The C-terminal peptides of different G proteins appear to assume many distinct orientations before being linked to their respective cognate GPCRs, as reported by Sivaramakrishnan and collaborators in 2013,^221^ with a live-cell FRET assay using Systematic Protein Affinity Strength Modulation (SPASM) sensor-based technology.^222^ This live-cell FRET assay did not need extensive purification of protein partners. The GPCR and G proteins were incorporated into giant plasma membrane vesicles (GPMVs) in a single step, and previously developed SPASM sensors were used, which enabled exploration of the interactions between GPCR and G protein in live cells.^221^ The assay was applied in β_2_AR in live cells complemented with MD simulations to describe the conformational ensemble reported by Vaidehi, Sivaramakrishnan, and collaborators in 2019.^223^ The results in ref 223 showed that the C-terminal peptides of Gαs, Gαi, and Gαq adopt a set of distinct orientations, in which, for example, the insertion angle of the principal axis of the C-terminus of the peptide with the principal axis of the GPCR is different when coupled to cognate GPCRs. This has been observed in the X-ray and cryo-EM structures of the corresponding complexes of G proteins with human GPCRs. In particular, based on the G protein preference of a GPCR, examples of experimental structures of such complexes are the following: Gαs-bound β_2_AR (PDB ID 3SN6 ^82^); mini-Gαs-bound A_2A_R (PDB ID 5G53 ^56^); Gαi-bound μOR (PDB ID 6DDE^149^);^149^ Gαi-bound A_1_R (PDB ID 6D9H^224^) reported by Christopoulos, Glukhova, Sexton, and collaborators in 2018;^224^ Gαo-bound to 5-HT_1B_R (PDB ID 6G79 ^225^) reported by Tate and collaborators in 2018;^225^ Gαi-bound to RhoR (PDB ID 6CMO^62^) reported by Xu, Subramaniam, Kossiakoff, and collaborators in 2018.^62^ Although noncognate GPCR–G protein interactions are important in cells, little is known about them. The study in ref 223 demonstrated that the noncognate G proteins dynamically interact with hidden, nonpreviously characterized GPCR cytosolic cavities, inducing the appropriate conformation for a subsequent coupling with cognate G proteins. Weak interactions between the noncognate G proteins and the intracellular GPCR cavities cause GPCR–G complexes to dissociate. Additionally, when G proteins engage with their cognate GPCRs, the C-terminus of Gαs, Gαi, and Gαq proteins can adopt a small dynamic set of distinct orientations, explaining the variations in their orientation observed in the X-ray and cryo-EM structures of GPCR–G protein complexes. Based on identified hotspot residues, which are used from the above-mentioned experimental structures of GPCRs for coupling with their relevant cognate Gαs or Gαi, or Gαq proteins, three predicted mutations in β_2_AR were shown to modify the hidden cytosolic cavity to bind effectively in a dose-dependent manner, also the noncognate Gαq protein and signal through both Gαs and Gαq. The tunability of G protein selectivity in GPCRs is demonstrated by this promiscuous triple mutant β_2_AR.

Using SPASM sensor-based technology, it was reported by Sivaramakrishnan and collaborators in 2021 ^226^ a two-stage activation mechanism of β_2_AR from cognate Gαs after agonist binding. Thus, interaction of the Gαs-CT peptide with an intermediate orientation of the cytoplasmic receptor part changes the receptor orientation to a full coupling conformation, which facilitates the engagement with the G protein and its full activation.^226^

##### 4.2.2.2 Kinetic Measurements

**a. Studies in Micelles**

**Dynamics of β**_**2**_ **AR**

According to ^19^F NMR studies of β_2_AR reconstituted in DDM/CHS micelles reported by Wüthrich and collaborators in 2013,^227^ significant structural reorganizations are necessary to achieve equilibria between the active and inactive states of the β_2_AR. This was demonstrated by an exchange rate in the sub-s time scale and an enthalpy difference (∼40 kJ mol^–1^) between the equilibrated states.

**I2** ^**TM6**^ conformation equilibrates with the inactive state (conformations **S1** ^**TM6**^, **S2** ^**TM6**^) on a slower time scale compared to the exchange between conformations **S1** ^**TM6**^ and **S2** ^**TM6**^, while **I2** ^**TM6**^ also equilibrates with **A** ^**TM6**^ conformation, as was shown by fluorescence spectroscopy in micelles by Kobilka and collaborators in 2004.^208^ The inactive conformations **S1** ^**TM6**^ and **S2** ^**TM6**^ in β_2_AR exist in slow exchange with active intermediate **I2** ^**TM6**^, as was revealed by the ^19^F solution NMR spectroscopy in MNG-3 micelles,^69,203^ or ^13^C solution NMR spectroscopy in DDM micelles.^91,194^ The ^13^C solution NMR studies in DDM micelles reported by Kobilka, Jin, and collaborators in 2013 ^91^ or Shimada and collaborators in 2013,^194^ showed an exchange between the inactive conformations and the **I2** ^**TM6**^ conformation on the ms time scale or longer. The smFRET combined with TIRF imaging results of full-length β_2_AR in DDM/CHS micelles also revealed an exchange between the inactive state (**S1** ^**TM6**^, **S2** ^**TM6**^) and active conformation **I2** ^**TM6**^ of the agonist-only bound β_2_AR and between **A** ^**TM6**^ and **I2** ^**TM6**^ conformations in the low-ms time scale.^97^ The ^19^F solution NMR studies of β_2_AR in MNG-3 micelles by Kobilka and collaborators in 2015 ^69^ showed that the inactive “ionic lock”-related conformations **S1** ^**TM6**^, **S2** ^**TM6**^ equilibrate between each other in a relatively fast NMR time scale (hundreds of μs), while the lifetime of the active **I2** ^**TM6**^ conformation was ∼600 ms.

Moerner and collaborators reported in 2011,^228^ using SMF on labeled β_2_AR at TM6 ends reconstituted in DDM micelles, movements of TM6 ends with durations of hundreds of ms when a full agonist binds the full-length β_2_AR. This was also shown by Gether and collaborators in 2001 ^229^ using fluorescence spectroscopy experiments with β_2_AR in DM micelles.

**Dynamics of β**_**2**_ **AR–G Complex Formation**

As previously mentioned, Blanchard, Kobilka, and collaborators in 2017 ^97^ performed smFRET with β_2_AR in DDM micelles with the receptor labeled with the FRET pair at cytoplasmic N148^4.40^C, L266^6.28^C in the presence of ligands of different efficacy, showing the effects on the kinetics and G protein coupling. According to the smFRET measurements, TM6 dynamics are nucleotide- and partial or full agonist-dependent, as reflected in measurements of two procedures that lead to Gs activation. Thus, it was measured: (a) the rate of formation of β_2_AR-Gs^GDP^ complexes and (b) the efficiency of GDP/GTP exchange.^97^ The apo-receptor, antagonist-, partial agonist-, and full agonist-bound β_2_AR–Gs states showed varying degrees of conformational heterogeneity, with increasing proportions of the active state resulting from higher-efficacy ligands. The interhelical distance distribution between TM4 and TM6 also varied according to ligand effectiveness in the G-bound complexes when the GDP was absent. While full agonists produce a primarily high-FRET population (active conformation with a closed cytoplasmic cavity and small TM4-TM6 distance), inverse agonists produce a predominantly low-FRET population (inactive conformation with an open cytoplasmic cavity and large TM4-TM6 distance); see Figure 5. Remarkably, it was shown^230^ that more efficacious ligands can enhance the probability of GDP release, which allows GTP binding with a higher rate and affinity to the agonist−β_2_AR–Gs^empty^ complex. These findings showed the allosteric connection between OBS and the GDP binding domain, as concluded in the work in ref 230. This was also shown by Ballet, Banères, and collaborators in 2025 in a study to characterize the interactions of ghrelin receptor (GHSR) with Gq using Gq peptidomimetics in combination with FRET and luminescence resonance energy transfer (LRET).^231^

**Figure 5:**
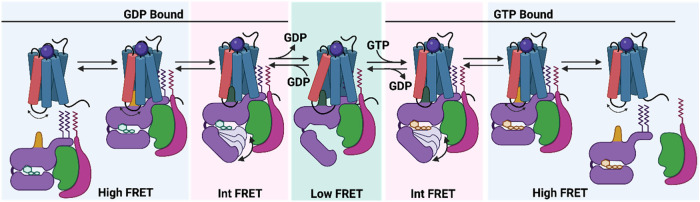
Schematic description of the conformations of TM6 during the activation of G protein in relation to FRET-based signaling; the low-FRET β_2_AR–Gs­(GTP) complex was deduced rather than seen experimentally (figure inspired by ref 97).

The results in ref 97 suggested the presence of relatively long-lived agonist−β_2_AR–Gs^GDP^ complexes during initial binding and, after G protein activation, long-lived β_2_AR–Gs^GTP^ complexes (Figure 6). Interestingly, it was suggested that the presence of preassembled complexes β_2_AR-Gs^empty^ and β_2_AR-Gs^GDP^ corresponds to a conformation **I1** ^**TM6**^ with low and intermediate FRET values compared to the higher FRET values in the ternary complexes with full agonists. These findings showed the different engagement of the α5 helix in these complexes. Such preassembled complexes were also suggested in ref 230.

**Figure 6:**
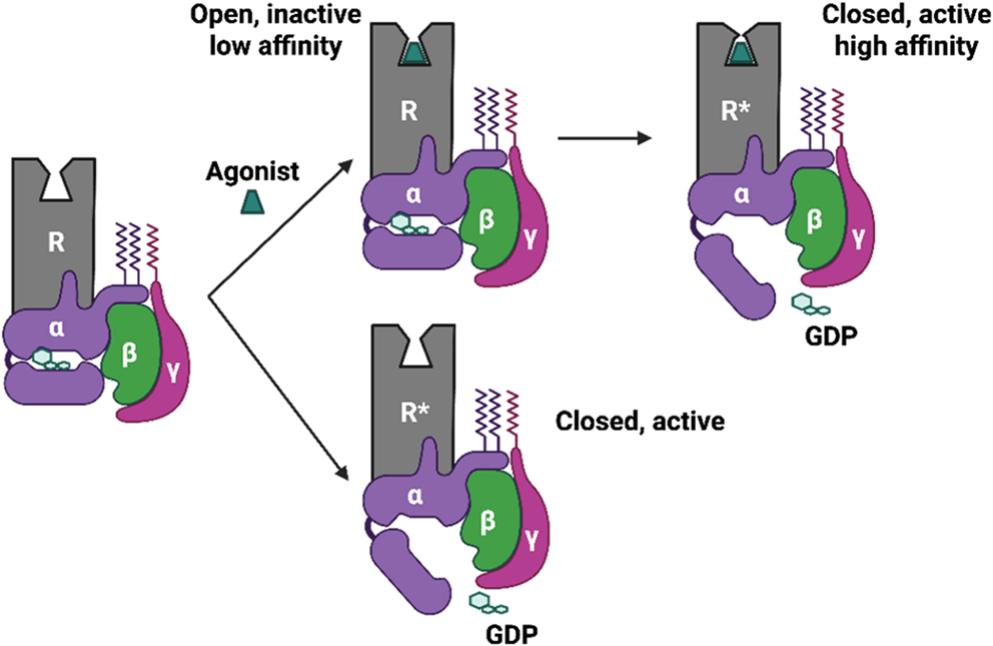
G-protein–receptor interaction and GDP release from the G-protein heterotrimer GαGβγ are facilitated by agonist binding. The C-terminal helix of Gα stays incorporated into the receptor core in this nucleotide-free state, maintaining the intracellular and extracellular conformational changes of the receptor. The affinity is increased at the extracellular side when the orthosteric ligand-binding site closes around the bound agonist, sterically opposing agonist dissociation. In the absence of an agonist, constitutive (basal) receptor activity may also activate the G protein, release GDP, and maintain the receptor’s closed conformation (figure inspired by ref 230).

**b. Studies in Lipid Bilayers**

**Dynamics of β**_**2**_ **AR**

SMF/TIRF microscopy in lipid nanodiscs^198^ showed that the inactive conformations **S1** ^**TM6**^ and **S2** ^**TM6**^ exist in slow exchange with the active intermediate **I2** ^**TM6**^, in agreement with the ^19^F solution NMR spectroscopy,^69,203^ or ^13^C solution NMR spectroscopy,^91,194^ or fluorescence spectroscopy,^208^ or smFRET^97^ in micelles. The SMF/TIRF microscopy enables measurements of longer-lasting movements compared to smFRET, and measured switches between inactive and active conformational states at the cytosolic TM6 end in a time range between 0.2 and 2 s.^198^ In the apo-form, the receptor adopts both conformations with a higher population toward the inactive state, while binding of the full agonist formoterol increases the frequency of activation transitions and favors the active-like conformation, and binding of the inverse agonist ISI-118,551 increases the frequency of deactivation transitions and shifts the equilibrium toward the inactive conformation.^198^

A subsequent SMF/TIRF study by Tutku, Stamou, and collaborators in 2025 ^232^ was performed with truncated β_2_AR at C365 reconstituted in liposomes to measure the dynamics of a single β_2_AR using a single small unilamellar liposome assay. It was shown^232^ that movements of TM6 end have a duration from long ms to min. In the apo-form, β_2_AR interconverts between states that are likely in the inactive region. Full agonist addition increases the population of states in the active region and slightly their duration, likely due to a conformational selection.

**Dynamics of β**_**2**_ **AR–G Complex Formation**

It has been demonstrated that active conformations (e.g., **A** ^**ΤM6**^, **I2** ^**ΤM6**^) promote GDP release by subsequently forming the more stable agonist–GPCR–Gs^empty^ conformation; thus, agonist–GPCR–Gs^GDP^ is an intermediate before the formation of the agonist–GPCR–Gs^empty^ complex. This was shown experimentally with BRET-based kinetics in cells in β_2_AR by Bouvier and collaborators in 2006,^233^ using β_2_AR- and Gβ- and Gγ-fused to BRET probes, e.g., to *Renilla reniformis* luciferase (Rluc) or other fluorescent proteins. Additionally, this was revealed with MS or crystal structures of β_2_AR performed by Chung, Kobilka, Lodowski, and collaborators in 2019,^26^ or by Kobilka and collaborators in 2019,^143^ respectively, and with MD simulations in β_2_AR performed by Shaw and collaborators in 2011.^209^

Sunahara and collaborators showed in 2016 ^230^ with radioligand kinetic binding experiments in lipid nanodiscs that the G protein coupling that causes the receptor’s basal activity hampers the association of agonists (e.g., agonist CGP12177 and full agonist formoterol), partial agonists (e.g., CGP-12177), antagonists (e.g., dihydroalprenolol), and inverse agonists (e.g., carvedilol). The G^empty^ only bound β_2_AR (in the absence of an agonist) stabilizes a closed conformation of the receptor with limited entry and exit of ligands from OBS and restriction of large conformational changes in the receptor (Figure 6). The structural reason for the G protein-mediated increase of agonist affinity, which has been noted for several GPCR–G-protein complexes, is that bound ligands are also prevented from dissociating from the receptor.

The smFRET dissociation measurements in ref 97 showed that the range of lifetimes of full agonist– or partial agonist−β_2_AR–Gs^empty^ complexes is 5–10 min. This lifetime was reduced when physiological amounts of GDP (30 μM) or GTP (100 μM) were added, by ∼20 to 100-fold, producing stable complexes for about 6–12 s. In the study, it was also observed that transient, precoupled β_2_AR-Gs^GDP^ complexes, after binding to an agonist or cellular partner, can undergo rapid allosteric, concerted motions.

Lambert and collaborators in 2018,^234^ using BRET experiments in live cells, showed that β_2_AR–mini-Gs complexes were quite stable when norepinephrine was present and needed more than 15 min to dissociate after treatment with the inverse agonist ICI 118,551. This is much slower than the few seconds of dissociation time of receptor–heterotrimer complexes in intact cells,^131^ and it likely reflects extra stabilization of the ligand binding in the OBS by the presence of mini-G protein mimicking the Gs^empty^ protein.

### 4.3 β_1_ Adrenergic Receptor

The conformational equilibrium in β_1_AR was investigated using ^15^N solution NMR of ^15^N-Val labeled receptor in micelles reported by Grzesiek, Veprinstev, Schertler, and collaborators;^235^ Grzesiek and collaborators;^236,237^ Abiko, Grzesiek, and collaborators^238^ between 2016 and 2020.^235–238^ The ^15^N amide signals of valine residues located near the extracellular surface were observed to follow the effect of ligands of different efficacy or the effect of adding an agonist and a G-protein-mimicking Nb. The equilibrated conformations, properties, and binding kinetics of β_1_AR using ^19^F of receptor labeled on TM6 (A282^6.27^C) and TM7 (C344^7.54^) cytosolic ends or ^13^C solution NMR spectroscopy based on Met at the cytosolic ends of TM5 and TM6 in micelles were reported by Nietlispach and collaborators between 2017 and 2024.^239–241^

The results from X-ray, cryo-EM, and NMR studies showed that, similar to β_2_AR and in contrast to A_2A_R,^69^ the conformational changes in TM5 and TM6, which are required for the full engagement of a G protein, are almost completely dependent on the presence of both the agonist and the G protein or mimetic Nb, revealing a weak allosteric coupling between the OBS and the G-protein-coupling interface (TM5 and TM6), with an intermediate active conformation of the agonist−β_1_AR complex being inactive-like. Thus, in ref 235 were observed changes in the ^15^N chemical shifts of valine residues located near the extracellular surface upon adding a G-protein-mimicking Nb in agonist−β_1_AR, in contrast to the ^15^N NMR data reported by Eddy and collaborators in 2021 ^155^ using the chemical shifts of extrinsic tryptophans located at the extracellular surface of the receptor.

The solution NMR studies showed that in the agonist sample, a conformational equilibrium exists between an inactive conformation, e.g., **S1** ^**TM6**^ (but not yet characterized), conformation **I2** ^**TM6**^, and conformation **A** ^**TM6**^. The population of **I2** ^**TM6**^ increased with enhanced agonist efficacy and reached 20% for isoprenaline. The solution ^19^F NMR study^239^ also identified **I1** ^**TM6**^ conformation as an activation intermediate. Addition of Gs or Nb6B9 to the isoprenaline-containing sample predominates the active conformation **A** ^**TM6**^ (that corresponds to the isoprenaline−β_1_AR–Gs complex), but also the **I1** ^**TM6**^ conformation, which corresponds to the β_1_AR–Gs complex, was observed. It was also shown that the time scale for conformational exchange between inactive and conformation **I2** ^**TM6**^ and between **I1** ^**TM6**^ and **A** ^**TM6**^ was in the slower sub-s time scale.^239^

### 4.4 μ Opioid Receptor

The conformational equilibrium of μOR in micelles was investigated with solution ^13^C NMR by Granier, Déméné, and collaborators in 2015 ^242^ using the resonances of ε-N­[^13^CH_3_] groups and with solution ^13^C NMR by Shimada and collaborators in 2015 ^243^ using the ^13^C-labeled Met in the cytoplasmic region. Additionally, Sounier, Granier, and collaborators in 2022 ^244^ used both ε-N­[^13^CH_3_] groups and ^13^C-labeled Met residues in the cytoplasmic region to study μOR with ^13^C solution NMR in micelles. The binding of peptide DAMGO and BU72 agonists, Gi-biased agonists oliceridine and PZM21, and the partial agonist buprenorphine.

Interestingly, the solution NMR data in refs 242,243 revealed that, like β_1_AR and β_2_AR, an intermediate-active state of agonist (e.g., BU72) in complex with μOR resembles the inactive state as regards conformation. This means that the movement of TM5, TM6, which are linked with the coupling of the GPCR with the G protein, is observed only when both the agonist and the G protein or mimetic Nb are present; no movement of TM5, TM6 is observed when only the agonist is present. However, more significant conformational changes were observed on ICL1 and H8 than on TM5 and TM6.^242,243^ These findings imply that one or both ICL1 and H8 domains may be involved in the first interaction with the G protein, while TM5 and TM6 are only involved later in the complex formation process. Additionally, that G-protein coupling selectivity may be influenced by these initial contacts between the G protein and ICL1 and/or H8, as has been proposed for other class A GPCRs, e.g., RhoR,^245^ M1R,^246^ or M3R.^247^

The results of the combination of ^13^C NMR and enhanced sampling MD simulations using the replica-exchange solute tempering (REST) method in ref 2022 ^244^ showed that Gi-biased agonists cause μOR conformational alterations in the ICL1 and H8 domains, which may hinder β-arr binding and signaling. Additional MD simulations toward this aim were performed by Goddard III and collaborators in 2019,^248^ and Scott, Shields, and collaborators in 2024.^249^

Elgeti, Kobilka, Chen, and collaborators used in 2024 ^250^ a combination of DEER and smFRET on μOR– Cy3/Cy5 and Cy3/Cy7 with labels at cytoplasmic TM4, TM6, respectively. Τhe DEER experiments, which inform on the conformation changes and dynamics of TM6, showed^250^ that the antagonist naloxone only weakly stabilizes one inactive cytoplasmic conformation, **S2** ^**TM6**^, over the inactive cytoplasmic conformation **S1** ^**TM6**^. Interestingly, with low-efficacy G protein-biased agonists, the TM4-TM6 distance remained mostly in the inactive **S1** ^**TM6**^ and **S2** ^**TM6**^ conformations, suggesting that other than TM6 cytoplasmic regions might modulate coupling with G protein, e.g., the H8 and ICL1 motifs suggested by NMR in ref 242. The smFRET data with Cy3/Cy7 fluorophore pair also showed a sub-ms exchange between the two inactive conformations **S1** ^**TM6**^ and **S2** ^**TM6**^ in samples with the antagonist naloxone and the low-efficacy agonists. Compared to the low-efficacy agonists, the binding of the high-efficacy agonist DAMGO caused a small but significant population shift (25%) toward the active conformations **A** ^**TM6**^ and **I2** ^**TM6**^ based on TM4-TM6 distance measurement, which, however, did not reflect the high efficacy (100%), suggesting again that structural changes other than the TM6 pivotal motion permit coupling with Gi. The superefficacy agonists BU72 and lofentanil stabilize only the two active conformations, **A** ^**TM6**^ and **I2** ^**TM6**^. The smFRET revealed only one active conformation present for the high-efficacy agonist DAMGO and the superefficacy agonist BU72. According to smFRET results, a slow exchange (>100 ms) between the inactive and active **I2** ^**TM6**^ conformation was observed with the smFRET on the μOR–Cy3/Cy7 construct. Since μOR–Cy3/Cy7 includes a labeling site close to the C-end of ICL2, the slow process can be due to a conformational motion of ICL2 or to the rotation of TM6 (the TM6 outward pivotal movement includes both translation and rotation of TM6), which is required for Gi coupling.^54^ The kinetics reported by Bünemann and collaborators in 2025 ^251^ using FRET- and BRET-based receptor conformation sensors (fluorescent proteins) showed strongly agonist-dependent activation kinetics of this receptor.

## 5 Mass Spectrometry

### 5.1 A_2A_ Adenosine and β_2_ Adrenergic Receptors

Various mass spectrometry techniques have been applied to GPCRs.^252^ HDX-MS has been used to investigate conformational changes and structural features related to the function of GPCRs.^26,253–255^ Chung, Kobilka, Lodowski, and collaborators studied in 2019,^26^ both A_2A_R and β_2_AR. Griffin and collaborators in 2010,^253^ Griffin and Stevens in 2011,^254^ Chung, Du, and collaborators in 2020 ^255^ studied β_2_AR. Thus, Griffin, Stevens, and collaborators explored in 2011 ^254^ with HDX-MS, the changes in conformations of β_2_AR reconstituted in DDM/CHS micelles induced by ligands of different efficacies. It was shown^254^ that the inverse agonists timolol and carazolol stabilize conformation in the cytoplasmic region where G protein couples with β_2_AR. They produce a more compact conformation compared with the antagonist alprenolol. The agonist isoproterenol induced the largest degree of conformational mobility, while the partial agonist clenbuterol produced conformational effects found in both the inverse agonists and the agonist. All the ligands induced conformations along the whole GPCR sequence that differ from the apo-form.^254^

Chung, Kobilka, Lodowski, and collaborators used^26^ a combination of HDX-MS and HRF-MS to describe a few distinct ICLs that undergo consecutive conformational changes in β_2_AR, and A_2A_R when they engage with the GDP-bound Gs protein. In detail, the sequence of steps that take place during GDP release mediated by GPCR was investigated in a time-resolved manner with time-resolved MS (HDX-MS and HRF-MS) applied in β_2_AR.^26^ When purified β_2_AR was mixed with Gs^GDP^, the complex isoproterenol−β_2_AR–Gs^GDP^ (PDB ID 6EG8 ^143^) was formed within a few seconds, as was shown by Liu, Kobilka, and collaborators in their structural study in 2019.^143^ Recall that the distance between the nucleotide-binding pocket and the interface between GPCR and G proteins is approximately 30 Å. Thus, the GDP release mediated by GPCR is achieved by conformational changes (allosteric) mediated by structural motifs between the GDP-binding pocket and the two proteins’ interface. It was further shown^26^ that conformational changes in β_2_AR by interaction of Gs with ICL2—with F139^ICL2^ (residue 34.51 in GPCRdb^78,126^ )—occur sooner than conformational changes in the N-terminus of ICL3. Thus, it was revealed that the large hydrophobic residue F139^ICL2^ in β_2_AR is not responsible for the initial contact with Gs but is crucial for causing the release of GDP from Gαs through interaction with the hydrophobic pocket formed by H41, V217, F219, and F376 in Gα (see discussion about the NMR findings on the conformations of cytoplasmic TM6 of A_2A_R).^26^ Mutation of F139^ICL2^ to alanine in the β_2_AR was detrimental for the release of GDP from Gs, although the F139^ICL2^A receptor could still contact Gs.^26^ The same was shown in a later HDX-MS study from Chung, Du, Inoue, and Ham in 2024 with the human muscarinic acetylcholine receptor M3 (M3R), revealing that L174^34.51^ was not crucial for the initial interaction between M3 and Gq but was crucial for the release of GDP from Gq.^256^ Importantly, the treatment of β_2_AR with agonist isoproterenol and Gs^GDP^ results in the formation of the crystal structure of the ternary complex within a few seconds.^143^ The HRF-MS studies showed^26^ the very slow rate of the conformational changes, including the cytoplasmic end of TM5 as well as the distal C-terminal Cα5 helix and the N-terminus of ICL3, which are needed for the formation of the nucleotide-free ternary complex agonist−β_2_AR–Gs^empty^, according to the HDX-MS results. Thus, the study showed^26^ that it takes 2–3 h for releasing GDP and forming the more stable agonist-β_2_AR–Gs^empty^. Additionally, interactions involved in the formation of a transient β_2_AR–Gs complex may be crucial in determining coupling selectivity.

Interestingly, all the class A GPCRs that have a secondary coupling also with Gi/o protein contain large hydrophobic amino acids at residue 34.51, suggesting that possibly residue 34.51 may be required for coupling with noncognate Gi/o proteins. However, while it has been shown that a bulky hydrophobic residue at 34.51 is important for the primary coupling of a GPCR with Gs protein^26,257^ (e.g., β_2_AR, A_2A_R) or with Gq/11 protein (e.g., M3R),^256^ residue at 34.51 may not be important for primary coupling of a GPCR, e.g., the μOR or M2R, with cognate Gi/o protein. Chung, Du, and collaborators in 2020 ^255^ explored comparatively with HDX-MS using as models GPCRs, the β_2_AR and M2R, the mechanism of coupling with Gi/o proteins as secondary (β_2_AR) or primary coupling (M2R), respectively, and explored the molecular determinants that differentiate primary coupling with Gs or Gi/o proteins, respectively. The results showed that residue 34.51 is also critical for the secondary coupling of β_2_AR to Gi/o but is not important to the primary coupling of M2R to Gi/o, and thus, it is still unclear what structural features allow the release of GDP during primary class A GPCR-Gi/o coupling. Indeed, available experimental structures of GPCR–Gi/o complexes revealed that hydrophobic residues at 34.51 are weakly bound through hydrophobic interactions with the hydrophobic pocket comprised by V34, L194/L195, F196/F197, and F336 of the Gαi/o proteins. Examples are the experimental structures of neurotensin receptor 1 (NTSR)–Gi1 complex (PDB ID 6OS9 ^123^) reported by Skiniotis, Kobilka, and collaborators in 2019, the μ-opioid receptor complex (μOR)–Gi1 (PDB ID 6DDE^149^) reported by Kobilka, Skiniotis, Manglik, and collaborators in 2018, or the M2-Go complex (PDB ID 6OIK^258^) reported by Skiniotis, Kobilka, and collaborators in 2019. The study showed that the C-end of Gαi/o differentiates primary and secondary Gi/o-coupling and provided some evidence that primary Gi/o-coupling might follow different molecular mechanisms compared to primary Gs coupling. The results also cast doubt on the “wavy hook’s” precise functional mechanism of the primary coupling with Gi/o.

Most HDX-MS and HRF-MS-based structural investigations track conformational changes of specific loops or N/C-terminal regions, and relatively few dynamics of TM domains are revealed. Compared to the information received for the loops alone from HDX-MS-based analysis, limited proteolysis coupled to mass spectrometry (LiP-MS) enables the simultaneous monitoring of conformational changes of many residues covering both ICL/ECL loops and TM domains of a GPCR. In the LiP-MS technique, the semitryptic peptide sequence would indicate the protease K (PK) cleavage site, which would represent the local protease accessibility associated with a conformational state/change. Shui, Xu, and collaborators applied LiP-MS in 2024 in A_2A_R reconstituted in DDM/CHS micelles.^259^ The binding of an antagonist to the receptor reduced PK accessibility in the ECL2 (e.g., residues L167^EL2^, F168^EL2^). For potent agonists, reduced accessibility was observed for the N^7.49^P^7.50^xxY^7.53^ motif (F286^7.51^, Y288^7.53^) at the cytoplasmic end of TM7, which undergoes a slight inward movement toward the receptor core for class A GPCRs upon agonist activation. Furthermore, LiP-MS revealed reduced accessibility at ICL2 upon antagonist binding, indicating a more rigid conformation of ICL2 compared to the apo-state, and increased accessibility upon agonist binding. The decreased accessibility of consecutive residues in ECL2 (L16^EL2^, F168^EL2^, E169^ECL2^) at the agonist Gs-coupled state compared to the Gs-uncoupled state revealed the allosteric effect of G protein coupling on increasing agonist affinity to the OBS. An enhanced accessibility of TM6 end (T224^6.26^) was measured at the agonist, Gs-coupled state vs the Gs-uncoupled state in agreement with the agonist-bound A_2A_R–mini-Gs complex structure, in which residue T224^6.26^ at the cytoplasmic TM6 end moves outward from the receptor core by ∼14 Å relative to the agonist-only bound A_2A_R structure.^260^

Akashi and collaborators applied nMS in 2023 ^261^ to detect the ternary complexes between β_2_AR, adrenaline, mini-Gs, and Nb80. There is a strong correlation between the β_2_AR–mini-Gs or β_2_AR–Nb80 complex ratio observed in the mass spectra and agonist/antagonist efficacy obtained using a cell-based assay.

### 5.2 β_1_ Adrenergic Receptor

Zenobi and collaborators, using nMS in 2019,^262^ reported that β_1_AR in the presence of the full agonist isoprenaline is complexed with Nb80 and mini-Gs with different active-like conformations. The complexes β_1_AR–Nb80, β_1_AR–mini-Gs are also formed in the absence of agonist, allowing for the quantification of the basal activity of β_1_AR. Additionally, it was followed by the disruption of the ternary β_1_AR–agonist-transducer complex by increasing the concentration of an inverse agonist, allowing for comparison of the ligands’ specific affinities for the β_1_AR.^262^ Complex formation in response to several ligands was examined. These ligands included full agonists (norepinephrine, carmoterol, and isoprenaline), partial agonists (dobutamine and salbutamol), and antagonists (cyanopindolol, carazolol, and carvedilol). Two partial agonists produced limited responses; however, all full agonists showed complete complex formation. The weak agonist cyanopindolol showed a very limited population of complexes, whereas the antagonists carazolol and carvedilol showed no discernible complex formation. The study can be extended to detect allosteric modulators, which should also affect complex formation. The multidimensional free energy landscape of the β_1_AR in both its apo- and adrenaline-bound states was explored with metadynamics and a set of “biologically motivated” collective variables (CVs) by Gervasio and collaborators in 2025; the method can be applied to other class A GPCRs.^263^

To investigate conformational features of ICL1-ICL3 that are engaged with activation of the receptor and to explore the efficacy of various ligands, nMS and HDX-MS in micelles were used as reported by Yen, Jazayeri, and Robinson in 2022 ^264^ and Robinson, Yen, and collaborators in 2024,^265^ while HDX-MS in micelles was reported by Politis, Hopper, and collaborators in 2024.^266^

The selectivity profile of β_1_AR to mini-Gs, mini-Gi, mini-Gq, or Nb was examined and reported by Ma, Zenobi, and collaborators in 2021 ^267^ using high-throughput matrix-assisted laser desorption/ionization MS (MALDI-MS). The β_1_AR binds even in the absence of agonist to its primary coupling partners, Gs and Gq, but also to some extent to Gi/o. Indeed, agonist-bound β_1_AR was observed to exist in multiple conformations that can be coupled with different transducer proteins. It was detected that formation of the β_1_AR–Gα_i_βγ or β_1_AR−β-arr1 occurred with or without the presence of ligand. Nb80 induces an allosteric effect that enables the displacement of the antagonist nadolol by isoprenaline.

The coupling selectivity of β_1_AR to Gs and Gi was tested and reported by Politis, Hopper, and collaborators in 2022 ^266^ using nMS by comparison of the binding of β_1_AR with mini-Gs and mini-Gi at equimolar ratios. The receptor’s high selectivity for the Gs protein was demonstrated by the 30% drop in the percentage of receptor-mini-Gi complex. Norepinephrine, isoprenaline, and carmotorol were examined separately to compare their capacities to promote Gi protein coupling. nMS made it evident that isoprenaline was more likely than the other two agonists to promote Gi protein coupling. Together, these results recapitulate the preference of the receptor, which couples primarily to Gs, with Gi and Go proteins serving as secondary transducers.

## 6 Effects of Lipids on Cytoplasmic Conformation

NMR spectroscopy has contributed to our current understanding of how biological membranes and lipids influence GPCR signaling, as reviewed by Jain and Eddy in 2025.^268^ It has been suggested that lipid nanodiscs containing cholesterol^269,270^ or docosahexaenoic acid^163^ shift the conformational equilibrium of the A_2A_R toward active conformational states, which increases the Gs protein activation; this shift was shown with a solution NMR of labeled A_2A_R at V229^6.31^C as reported by Shimada and collaborators in 2020,^163^ or Prosser and collaborators in 2022,^269^ respectively. The population of the A_2A_R active conformational state was considerably increased by direct interactions with cholesterol and cholesterol analogs when anionic lipids were not present, as was shown with solution ^19^F NMR spectroscopy of A_2A_R in lipid nanodiscs by Eddy and collaborators in 2023.^271^ However, cholesterol had a negligible effect on A_2A_R activation when anionic lipids were present, indicating that cholesterol’s effect could be more indirect under these circumstances, which is consistent with findings reported by Prosser and colleagues in 2022 ^269^ who also used solution ^19^F NMR spectroscopy in lipid nanodiscs containing anionic lipids.

Eddy and collaborators showed in 2023 ^272^ using ^19^F solution NMR of labeled A_2A_R at A289^7.54^C in lipid POPC/POPS nanodiscs that anionic phospholipids, for example, phosphatidylinositol-4,5-bisphosphate (PI­(4,5)­P2 or PIP2), enhanced the population of active-like conformation **A** ^**TM6**^, thus priming the receptor toward recognizing Gαs partner protein and signaling. The effect of the anionic phospholipids toward the formation of the active-like conformation of A_2A_R for coupling with G proteins has been shown using nMS by Robinson and collaborators in 2018,^273^ while Sansom and collaborators predicted interaction with cytoplasmic residues of TM6 and TM7 of A_2A_R through CG MD simulations in 2019.^274^

STD NMR in the β_2_AR under lipidic cubic phase (LCP) conditions, conducted by Milon, Chezerov, and collaborators in 2014,^275^ revealed that β_2_AR interacted with cholesterol but not with the structurally related chemical ergosterol, indicating that GPCR-cholesterol interactions were unique to β_2_AR. Abiko, Grzesiek, and collaborators in 2022,^238^ using ^15^N-Val labeled β_1_AR, studied the impact of the cholesterol analog CHS. Without CHS, increasing pressure caused the β_1_AR conformational equilibrium to move toward an active conformation, according to two-dimensional TROSY NMR spectra of the protein. The addition of CHS prevented pressure-induced β_1_AR activation. These results support the notion that CHS functions as a negative allosteric modulator of β_1_AR signaling. The above-mentioned data suggested that various GPCRs may be impacted by cholesterol and cholesterol analogs in remarkably varied ways, even within class A receptors.

## 7 Discussion

### 7.1 Anticipated Findings from Exploring the Conformational Diversity of Class A GPCRs

The identification and structural characterization of the conformational equilibrium and the kinetics of the conformational changes of class A GPCRs are required toward understanding and controlling signaling selectivity and agonist efficacy. A conformational energy landscape including many transient conformers in the inactive and active regions is characteristic of the multistate nature of GPCRs. These distinct conformations^2–8,276–280^ are allosterically connected^40,42^ through a conserved canonical “microswitch” motif network^3,22–24,45^ and different energy barriers. A ligand can affect the equilibrium of these transient conformational states of a GPCR, boosting the predominance of some with low population or even permitting the presence of previously inaccessible conformations in the apo-state and restricting access to others. Compared to the endogenous agonist, various synthetic agonist-specific active states of a GPCR might result in unique activation conformations and signaling patterns through coupling with distinct transducer proteins (G proteins or β-arrs or GRKs), producing a different set of physiological responses. Femtoseconds (fs) to seconds (s) make up the time frame of motion that is relevant to GPCR function.^281^ Functionally significant dynamics are characterized by procedures such as the fs-ns photoexcitation and retinal isomerization in RhoR (chemical bond motions),^282^ ns-ms toggling of microswitch motifs (parts of TM helices, loops, amino acid residue side chains),^91^ ms exchange between functional states of the receptor,^68^ and activation of the G protein in the ms-s time scale.^97^ Additional cellular processes that can further regulate the strength and duration of signaling include expression and trafficking, phosphorylation, and then recruitment of β-arr, followed by desensitization and internalization. The membrane environment of the receptor can also affect signaling.

Research in this field sought to explain the following critical issues for class A GPCRs:

(a) How each conformation affects the different downstream signaling pathways through coupling with cognate or noncognate G proteins (primary or secondary G protein coupling, respectively) or β-arr. Additionally, how bound ligands can differentially stabilize the corresponding signaling complexes,^45^ as well as how to rationally develop “biased” or “functionally selective” ligands that elicit a desired mode of GPCR signaling.^37,72–75^

  Thus, different active conformations of a GPCR can have different affinities for ligands and transducer proteins, making a full agonist in a ternary complex with a G protein, a partial agonist in a complex with a β-arr, or vice versa, as is the case with carvedilol against β_2_AR for Gs and β-arr (see discussion in the last section of the review). The identification of ligands that can variably modify signaling pathways mediated by a single GPCR enhances their therapeutic value and represents a potential area in drug development. This is because such ligands can bind distinct conformations and activate favorable pathways while inhibiting deleterious ones.^37,72–75^ Additionally, as reviewed by Zhang and Lu in 2019,^283^ or López-Rodríguez and Vázquez-Villa in 2020,^284^ allosteric modulators can stabilize distinct conformations, promoting the activation of a particular signaling pathway.
  Data becoming available from X-ray and cryo-EM structures,^76,285^ solution NMR studies,^77^ and other biophysical studies can contribute toward this aim. For example, compounds that bind to the receptors and are helpful tools for laboratory investigations have already been discovered thanks to several class A GPCR structures and physical screening methods, as has been reviewed by Roth and Shoichet in 2017.^286^
(b) The interpretation of the basal activity of GPCRs due to a population of a conformation in the active region of the conformational landscape in the apo-state, explored, e.g., by Kobilka and collaborators in 2009 using FRET of β_2_AR labeled at the TM6 end.^287^
(c) The characterization of the important transient conformations and their changes in cells, and the understanding of the role they play in signaling. Τhe impact of agonist-free GPCR–G protein complexes and the conformation of the corresponding GPCR in the pharmacological response is not well understood.^36^

Results as regards the conformational landscape for the A_2A_R, β_2_AR, and β_1_AR have been included in review articles by Nietlispach and collaborators, Prosser and collaborators, Eddy and collaborators, or Ye and collaborators, between 2019 and 2023, see refs 8,165,271,276,277,288 or Gooley, Scott, and collaborators,^289^ while more general reviews about the conformational heterogeneity of GPCRs can be found in refs 1–8 . Here, we reviewed important observations for A_2A_R, β_2_AR, β_1_AR, and μOR.

### 7.2 Key Findings from Research on the Conformational States of A_2A_ Adenosine, β_2_ and β_1_ Adrenergic, and μ Opioid Receptors

The successful coupling of a class A GPCR to a G protein requires that the canonical “microswitch” motif network be composed of the following motifs: C^6.47^W^6.48^P^6.50^ (CWxP),^46^ the P^5.50^I^3.40^F^6.44^ (PIF),^47^ Na^+^ pocket,^48^ D^3.49^R^3.50^Y^3.51^ (DRY),^49^ N^7.49^P^7.50^xxY^7.53^ (NPxxY)^50^ along with the conserved Y^5.58^ residue in the T^3.46^Y^5.58^Y^7.53^ motif. Compared to the inactive conformation, this included the inward movement of residues I^3.40^ from the PIF motif and R^3.50^ from the E/DRY motif that pushes F^6.44^ (PIF motif) and L^6.34^ outward, resulting in the outward displacement of the cytoplasmic portion of TM6 from TM7. Simultaneously, the intracellular part of TM5 swings outward, while its central and EC segments shift inward by ∼2.5 Å. A key step in activation is the downward movement of Y^5.58^ in TM5, enabling a water-mediated hydrogen bond with Y^7.53^ of the NPxxY motif^50^—forming the so-called “YY-lock” (Y^5.58^---W^6.48^---Y^7.53^ or Y^5.58^---water---Y^7.53^). In contrast to the inactive conformation, this allosteric network stabilizes an outward swing of TM6 by ∼7–14 Å, generating a cytoplasmic cavity for Gα protein binding, as demonstrated in crystallographic, biophysical, and biochemical studies.^62,93^ Additionally, the conformational shift of W^6.48^ (“toggle switch”) further facilitates this process. In the agonist-only bound state, TM5 and TM6 α-helices remain highly dynamic to mediate this pronounced conformational change, observed in numerous class A GPCRs, that enables coupling to G proteins.

While A_2A_R, β_2_AR, and β_1_AR are engaged through interactions of cytoplasmic TM5, TM6 with Gs protein, in μOR, bigger ICL1 and H8 domains are involved in the recognition with Gi/o protein and showed significant conformational changes upon treatment with Go protein, while TM5 and TM6 are only involved later in complex agonist−μOR–Gi/o^empty^ stabilization. This was shown using solution NMR^242,243^ as well as DEER and smFRET.^250^

The remarkable sensitivity of the ^19^F chemical shifts to changes in the noncovalent environment is one of the benefits of ^19^F NMR probes. In this area of research, it was shown that ^19^F NMR markers are appealing reporter groups when examining function-related conformational equilibria and rate dynamics in membrane proteins. However, it has been noted that there are cases where ^19^F chemical shifts were not sensitive to conformational alterations that have been identified by other techniques in GPCR research. Interestingly, in ref 290, Wüthrich and Liu reported that all ^19^F labeling sites that displayed conformational changes are situated close to aromatic residues, according to an analysis of previously published ^19^F NMR data on the β_2_AR and mammalian RhoR.

While the formation of the agonist isoproterenol−β_2_AR–Gs^GDP^ complex takes a few seconds,^143^ the HRF-MS studies showed^26^ the very slow rate (2–3 h) of the conformational changes, including the cytoplasmic end of TM5 as well as the distal C-terminal Cα5 helix and the N-terminus of ICL3, which are needed for releasing GDP and forming the more stable agonist-β_2_AR–Gs^empty^. In contrast, the study found that it takes 2–3 h for releasing GDP and forming the more stable agonist−β_2_AR–Gs^empty^.^26^ Therefore, the agonist–GPCR–Gs^GDP^ complex is an intermediate before the formation of the agonist–GPCR–Gs^empty^ complex, as was shown experimentally with BRET-based kinetics in cells in β_2_AR and reported by Bouvier and collaborators in 2006.^233^ Additionally, interactions involved in the formation of a precoupled β_2_AR–Gs complex (with **I1** ^**TM6**^ conformation) may be crucial in determining coupling selectivity. It was also shown with ^19^F NMR in micelles^97^ that the rate of formation of β_2_AR-Gs^GDP^ and the efficiency of GDP/GTP exchange are increased according to the ligand’s efficacy, showing the allosteric connection between the nucleotide-binding pocket and the GPCR-G interface (separated by 30 Å) as well as between OBS and the nucleotide-binding pocket. The presence of preassembled β_2_AR–Gs^empty^ and β_2_AR–Gs^GDP^ complexes is mechanistically important, showing pathways for the assembly of the ternary complexes in which the agonist binds last in sequence.^97^

Research combining solution NMR, SMF, smFRET, and DEER, often complemented by MD simulations, has shown that class A GPCRs are an ensemble of conformations that are equilibrated according to their stability and energy barriers for their interconversion. The different minima in the conformational landscape correspond to multiple inactive and active transient states. Solution NMR and DEER spectroscopy have been used toward the identification of the number of conformations and characterization of their identity. smFRET helps study the kinetics of exchange between conformations, while SMF can detect processes with much higher or very slow rates.

The results showed that the free energy surface as regards the movements of the TM6 in A_2A_R and β_2_AR includes two inactive conformations (**S1** ^**TM6**^ and **S2** ^**TM6**^) and three conformations **I1** ^**TM6**^, **I2** ^**TM6**^, and **A** ^**TM6**^ in the active region of the conformational landscape, while **I2** ^**TM**6^ and **A** ^**TM6**^ conformations are, correspondingly, low-efficacy and high-efficacy activation states, reinforced by full agonist and partial agonist. In A_2A_R or β_2_AR, **I2** ^**TM**6^ and **A** ^**TM6**^ conformations correspond to noncognate Go^empty^ or Gi^empty^ and cognate Gs^empty^ proteins, respectively. The fully activated-like conformation of **A** ^**TM6**^ has been suggested^154^ to be like the fully activated conformation observed in the experimental structure agonist–GPCR–G. In A_2A_R, there is a strong allosteric connection between the ligand-bound OBS and the cytoplasmic region where the G protein binds, in contrast to β_2_AR, as has also been suggested by MD simulations of β_2_AR by Shaw and collaborators in 2011.^209^ In A_2A_R, conformation **A** ^**TM6**^ has a PIF conformation similar to the fully activated conformation in the experimental structure NECA–A_2A_R–mini-Gs complex (PDB ID 5G53 ^56^) or the cryo-EM structure agonist adenosine–A_2A_R–Gαβγs^empty^ complex (PDB ID 6GDG^85^) while **I2** ^**TM6**^ might correspond to the X-ray structure of the agonist NECA or adenosine-only bound A_2A_R (PDB ID 2YDV^150^ or 2YDO,^150^ respectively). Similarly, in β_2_AR, **A** ^**TM6**^ corresponds to the fully activated conformation observed in the X-ray structures agonist BI-167107−β_2_AR–Gαβγs^empty^ complex (PDB ID 3SN6 ^82^) and agonist BI-167107−β_2_AR–Nb6B9 complex (PDB ID 4LDE^138^).

Interestingly, the cryo-EM structure of an adenosine–A_2A_R–mini-Gαsβγ complex^80^ with A_2A_R bearing the mutation R291^7.56^A (PDB IDs 9EE8, 9EE9, 9EEA^80^) showed that this receptor adopts the **I2** ^**TM6**^ conformation. The GaMD simulations experimental assay^55^ further confirmed that A_2A_R adopts a nearly fully activated intermediate state.^80^ Thus, the activation intermediate adenosine–A_2A_R-Gαβγs complex in the **I2^ΤM6^** conformation can bind GDP but lacks the property of an efficient GDP/GTP exchange. This work addresses a knowledge gap in the intricacy of class A GPCR signaling.

The PRE ^15^N solution NMR experiments showed^162^ that the PIF motif and TM6 cytosolic conformation in **I2** ^**TM6**^ and **S1** ^**ΤM6**^ conformations are similar according to the weak coupling between the OBS and cytoplasmic region in conformation **I2** ^**TM6**^. The ^15^N NMR results showed that β_2_AR **I2** ^**TM6**^ has a PIF conformation like the conformation in the X-ray structure of the agonist carmoterol−β_2_AR complex (PDB ID 2Y02 ^161^) or the inverse agonist ICI 118,551−β_2_AR complex (PDB ID 3NY8 ^67^) or the antagonist alprenolol−β_2_AR complex (PDB ID 3NYA^67^).

Precoupled state **I1** ^**TM6**^ is a very interesting activation intermediate that corresponds to a GPCR–G complex. The research in A_2A_R showed that the populations of the TM6 activation states (**I1** ^**TM6**^, **I2** ^**TM6**^, **A** ^**TM6**^) and TM7 activation states (**I1** ^**TM7**^, **I2** ^**TM7**^, **A** ^**TM7**^) may be correlated, albeit not identical. In β_1_AR, an uncharacterized inactive conformation (that can correspond, e.g., to **S1** ^**TM6**^), and active conformations **I1** ^**TM6**^, **I2** ^**TM6**^, and **A** ^**TM6**^ were observed, and in μOR were observed the inactive conformations **S1** ^**TM6**^ and **S2** ^**TM6**^, and conformations **A** ^**TM**6^ and **I2** ^**TM6**^.

^19^F solution NMR^196^ or ^1^H NMR based on the TMS group^291^ of the β_2_AR in DDM/CHS micelles labeled at the cytoplasmic ends of TM6 (C265^6.27^) and TM7 (C327^7.54^) or SMF/TIRF microscopy^198^ showed that a Gs-biased agonist, for example, isoproterenol, perturbs the conformation of the cytoplasmic TM6 end, a β-arr-biased agonist, for example, isoetharine, perturbs TM7 cytoplasmic end, while a balanced agonist, for example, formoterol, perturbs both cytosolic TM6 and TM7 ends. Thus, ligands have the innate ability to alter the receptor’s conformational exchange kinetics, causing signaling bias. This is a novel and additional criterion that should be considered in drug-discovery initiatives (see discussion in the last section of the review).

The exchange between inactive and active states at the cytoplasmic TM6 of A_2A_R using ^19^F solution NMR spectroscopy in micelles,^68,153^ or smFRET in micelles and lipid nanodiscs,^182^ and the exchange between **I2** ^**ΤM6**^ and **A** ^**ΤM6**^ conformations at the cytoplasmic TM6 in the apo- and partial agonist- or full agonist-bound states using smFRET micelles^154^ occurs in the low-ms time scale. A study with ^15^N solution NMR in micelles showed that the exchange rate between the inactive and active states at the cytoplasmic TM6 of A_2A_R in micelles was slower than 20 ms, and between active states (at least two) having different conformation of the NPxxY motif is in a faster rate of exchange than the 20 ms scale.^163^ The exchange between **S1** ^**TM7**^ and **I1** ^**TM7**^ conformations at the cytoplasmic TM7 of A_2A_R using ^19^F solution NMR spectroscopy in lipid nanodiscs was in the sub-ms time scale.^167^

The ^13^C solution of β_2_AR NMR in micelles^91,194^ also showed exchange between the inactive conformations and **I2** ^**TM6**^ conformation on a ms time scale or longer. The smFRET/TIRF in micelles also revealed an exchange in the low-ms time scale between the inactive state and **I2** ^**TM6**^ and between **A** ^**TM6**^ and **I2** ^**TM6**^ conformations.^97^ The SMF/TIRF microscopy in lipid nanodiscs^198^ agreed with the results in micelles. The time scale for conformational exchange between inactive to conformation **I2** ^**TM6**^ and **I1** ^**TM6**^ to **A** ^**TM6**^ is in the sub-s time scale according to ^19^F NMR in micelles.^239^ Indeed, the ^15^N experiments in ref 162 revealed that **S1** ^**ΤM6**^ and **I2** ^**TM6**^ conformations are in exchange, while, compared to **S1** ^**ΤM6**^ and **I2** ^**TM6**^ conformations, **A** ^**TM6**^ conformation exhibits a major TM6 outward pivotal movement associated with a large conformation change in the PIF motif.^162^

smFRET experiments showed a sub-ms exchange between the two inactive conformations **S1** ^**TM6**^ and **S2** ^**TM6**^ in μOR and a particularly slow exchange (>100 ms) between the inactive state and active **A2** ^**TM6**^ conformation. This slow process can be a conformational motion of ICL2 or the rotation of TM6 in its pivotal motion required for coupling to Gi.^54^

In structures of signaling complexes of class A GPCRs that couple to a Gi protein, the outward movement of TM6 is smaller compared to Gs and Gq/11 complexes because of the smaller size of the Gαi protein binding pocket in the cytoplasmic core of the receptor compared to the Gαs and Gαq proteins. This is shown, for example, by comparison of the X-ray structure of BI-167107−β_2_AR–Gs complex (PDB ID 3SN6 ^82^), and the cryo-EM structure of the agonist peptide DAMGO−μOR–Gi1 complex (PDB ID 6DDE^149^). Equivalently, this corresponds to a smaller size of the C-terminus of the Cα5 helix of Gαi compared to the bulkier Cα5 helix of the Gαs and Gαq proteins, in agreement with the HDX-MS results.^255^

It must be underlined that there is a concern regarding the consistency of results from the HDL system in lipid nanodiscs with pharmacological research. Thus, the percentage of conformations in the active region of the conformational landscape was observed for both A_2A_R^292,293^ and β_2_AR^294^ using ^19^F NMR, abnormally high compared to the micelles system, e.g., MNG-3/CHS. For example, in ref 294, β_2_AR contains over 40% activation conformations by solution ^19^F NMR in lipid nanodiscs, in contrast to the low constitutive activity of the receptor. Even though only some GPCRs, including the A_2A_R and β_2_AR, have had their conformational states profiled by ^19^F NMR studies, it seems that the HDL system may not be appropriate, possibly because the scaffold protein MSP1D5 may affect the chemical shifts, perturbing its conformational profile. The HDL system may be better for studying lipid effects on receptor activation and certainly on obtaining structures of the fully activated complex with G proteins using cryo-EM.

A combination of HDX-MS and HRF-MS revealed^26^ that in β_2_AR and A_2A_R, conformational changes that include an interaction of Gs with ICL2 occur sooner than conformational changes in the N-terminus of ICL3. It was further shown^26^ that the large hydrophobic residue F139^ICL2^ (F^34.51^) in β_2_AR is not responsible for the initial contact with Gs but is crucial for causing the release of GDP from Gαs through interaction with the hydrophobic pocket in Gαs, as discussed previously.^26^ The same was shown in another HDX-MS study with the human muscarinic acetylcholine receptor M3 (M3R), revealing that L174^34.51^ was not crucial for the initial interaction between M3 and Gq but was crucial for the release of GDP from Gq.^256^ HDX-MS showed that a bulky hydrophobic residue at 34.51 is important for the primary coupling of a GPCR with Gs protein^26,257^ (e.g., β_2_AR, A_2A_R, β_1_AR) and with Gq protein (e.g., M3R)^256^ and secondary coupling with Gi/o proteins (β_2_AR, β_1_AR), but is not important for primary coupling of a GPCR, e.g., the μOR, or M2R, with cognate Gi/o protein.^255^ It is still unclear what structural features allow the release of GDP during primary class A GPCR-Gi/o coupling. Experimental structures of GPCR–Gi/o complexes^123,149,258^ revealed that hydrophobic residues at 34.51 are weakly bound through hydrophobic interactions with the hydrophobic pocket of the Gαi/o proteins. MALDI-MS showed that β_1_AR binds even in the absence of agonist to its primary coupling partners, mini-Gs and mini-Gq, but also to some extent to the mini-Gi/o.

NMR spectroscopy has contributed to our current understanding of how biological membranes and lipids influence GPCR signaling, as reviewed by Jain and Eddy in 2025.^268^ It has been shown with ^19^F solution NMR of V229^6.31^C labeled A_2A_R that when cholesterol is added to lipid nanodiscs that are devoid of anionic lipids,^269–271^ it shifts the conformational equilibrium of the A_2A_R through direct interactions toward active conformational states, which increase the Gs protein activation. In contrast, cholesterol seems to act as a NAM against β_1_AR signaling since, according to ^15^N NMR spectra of the ^15^N-Val labeled β_1_AR, the absence of CHS moves the conformational equilibrium of β_1_AR toward an active conformation. These data suggested a divergent role of cholesterol and cholesterol analogs against the signaling of class A GPCRs. According to ^19^F solution NMR of A289^7.54^C labeled A_2A_R^272^ or nMS,^273^ supported by CG MD,^274^ anionic PIP2 phospholipids enhanced the population of active-like conformations, priming the receptor toward recognizing Gαs partner protein and signaling.

### 7.3 Research with Therapeutic Relevance

The NMR work described enabled the development of assays targeting GPCRs through new ligands.^77,288^ Interestingly, Ye and collaborators in 2022 ^295^ developed an in situ solution-state NMR approach (WaterLOGSY) to investigate high-throughput ligand–receptor interactions for faithful ligand screenings through homogenizing membranes embedded with native A_1_R receptors, which can also be applied to other GPCRs. The method enables the screening of more than 1,000 compounds using only 250 mL of cell culture. In the same context, Eddy and collaborators in 2025 ^296^ developed an in situ methodology that was able to screen fragment molecules with *K*_d_ > 1 μM against unpurified mg of cell membranes containing ∼1 μM of A_2A_R based on ^1^H MAS ssNMR in combination with STD NMR.

The design of drugs that specifically target a conformation linked to a disease will be made easier if we comprehend the roles that various conformational states and their complexes play in signaling bias. The topic of biased signaling through G-proteins or β-arrs and its relevance to drug design was reviewed in 2024 by Baidya, Kumari, and collaborators.^297^

β_2_AR is a prototype receptor for which ligands with varying propensities activate distinct pathways.^298^ β_2_AR couples with high selectivity to Gs and less Gi proteins. It is important to understand the molecular details responsible for the promiscuous coupling signaling through both Gαs and Gαi proteins, since the Gi signaling pathway may be related to heart failure.^299^ Thus, although β_2_AR agonists have as their main therapeutic target the Gαs pathway, in the heart tissue, β_2_AR couples to Gαi. The Gαi signaling pathway may be related to heart failure, as was reported by Bernstein and collaborators in 2013.^299^

It is important to understand the molecular details between biased agonists for Gs vs Gi-mediated signaling. In this context, Kobilka, Lerch, Gmeiner, and collaborators reported in 2024 ^211^ the cryo-EM structure of the complex agonist LM189−β_2_AR–Gi (PDB ID 9BUY^211^). Salmeterol is a β_2_AR partial agonist for Gs coupling and a full agonist for the recruitment of Gi with an efficacy greater than the native agonist epinephrine, as shown in ref 300. In comparison to salmeterol, which prefers signaling through Gi protein compared to Gs coupling, in ref 211, the full biased-Gi agonist LM189 was discovered. As expected, the OBS of the β_2_AR in the experimental structure of the LM189−β_2_AR–Gi complex is like the OBS of β_2_AR in the X-ray structure of β_2_AR–salmeterol complex or of β_2_AR–epinephrine complex (PDB IDs 6MXT^300^ or PDB ID 4LDO,^138^ respectively). However, while LM189 bears the long chain of salmeterol, the saligenin group is replaced by the catechol group in epinephrine. Thus, compared to salmeterol, OBS LM189 forms stable hydrogen bonds with S203^5.42^ and S207^5.46^, but also with N293^6.55^, while N293^6.55^ also forms a stable hydrogen bonding network with Y308^7.35^ and S203^5.42^. It seems that the mechanism of the specificity for Gi by LM189 might be due to intermediate conformations of the receptor that involve the TM core and the cytoplasmic cavity of the receptor. The combination of smFRET that monitors TM6 dynamics and DEER that monitors the TM4-TM6 distance in ref 211 suggested that the Gαi bias of LM189 compared to unbiased agonists, e.g., BI-167107, is due to a change in the conformation and dynamics of ICL2 and cytoplasmic TM6. It was shown^211^ that the Gαi-biased LM189, compared to the biased Gαs agonist epinephrine, stabilizes a distinct conformation in TM6 and increases the dynamics of ICL2. The DEER studies showed that LM189 stabilizes the cytosolic TM6 conformation when coupled to Gi compared to Gs. Solution NMR studies^200^ and HDX-MS^26,257^ discussed previously have shown that ICL2 of β_2_AR, with a tight α-helix in the case of Gs, compared to a loose conformation in Gi, binds more tightly with Gs compared to Gi. The selectivity of a signal transducer protein may have therapeutic benefits, and current drug research is heavily focused on exploring this notion.

The conformational space for binding to GRKs is understudied. β_2_AR agonists are considered poor clinical candidates for glycemic management due to Gs/cAMP-induced cardiac side effects and β-arr-dependent desensitization. Bengtsson, Wright, and Volker, and collaborators in 2025,^301^ using ligand-based virtual screening and chemical evolution, they developed pathway-selective agonists of β_2_AR that prefer GRK coupling. These pathway-selective agonists of β_2_AR that prefer GRK coupling can offer potential for the treatment of metabolic diseases such as type 2 diabetes and obesity.

It is believed that the μOR-mediated activation of a Gi protein is what gives opioid drugs like morphine their painkilling effects, while the receptor’s coupling to β-arr is what gives the drugs their addictive properties and often the lethal suppression of respiratory functions. The first study performed showing that β-arr signaling has implications on the in vivo pharmacological effects of drugs was the μOR desensitization by β-arr2 that determines morphine tolerance but not dependence, published by Caron and collaborators in 2000.^302^ Afterward, the design and synthesis of opioid molecules that relieve pain while lowering the danger of addiction or overdose has been attempted; see, e.g., the structure-based drug design work toward this aim reported by Shoichet, Roth, Gmeiner, Kobilka, and collaborators in 2016.^13^ Additionally, the development of bitopic agonists such as fentanyl-guanidinum conjugates targeting both the OBS and water-rich Na^+^ site,^303^ as was shown in cryo-EM structures (PDB IDs 7U2L, 7U2K^304^) provided a way to modulate the efficacy and functional selectivity profile for Gi, Go, and Gz subtypes and arrs in the in vivo pharmacology as reported by Majumdar, Kobilka, Katritch, Skiniotis, and collaborators in 2022.^304^ This approach led to a biased agonist for Gi recruitment over β-arr, as reported by Majumdar, McLaughlin, Hüttenhain, Wang, and collaborators in 2024 ^305^ or Majumdar and collaborators in 2025.^306^ Oliceridine, a partial agonist that binds at the OBS of μOR, was approved by the FDA in 2020 for its ability to biased signaling via the G protein pathway and thus alleviate side effects. Although progress has been made with structures and biased ligands against β_2_AR,^211,300^ μOR, and other class A GPCRs,^307^ the level of progress for A_2A_R is low.^308,309^ Allosteric ligands provide therapeutic potential through binding and stabilization of certain conformations.^310^

Overall, understanding and harnessing the signaling of GPCRs to develop drugs is a complicated task.^75,77,278,288,296^ Since the “quality” of drug efficacy, at least for GPCRs, is now known to encompass much more than the activation of widely assessed pathways like cAMP signaling, with many “efficacies” of ligands useful therapeutically, deeper characterization is becoming increasingly significant.^311^ Novel assays for describing the complicated behavior of GPCRs, such as biased signaling and allosteric regulation, as well as developments in structural biology, can make this kind of characterization possible. The molecular determinants of G protein or β-arr coupling specificity are still not accurately defined, despite many available experimental structures of class A GPCRs in combination with different G protein subtypes.^297^

## Abbreviations

A_2A_R: adenosine A_2A_ receptor
AC: adenylyl cyclase
AFM: atomic force microscopy
AWH: accelerated weight histogram
AHD: α-helical domain
arr: arrestin
AngII: angiotensin II
β_1_AR: β_1_ adrenergic receptor
β_2_AR: β_2_ adrenergic receptor
biotinyl CAP PE: POPE-N-(cap biotinyl)
BRET: bioluminescence resonance energy transfer
CHS: cholesteryl hemisuccinate
CAM: constitutively activating mutations
CNS: central nervous system
cryo-EM: cryogenic electron microscopy
CWxP: C^6.47^W^6.48^P^6.50^
DDM: dodecyl-β-D-maltoside
DEER: electron–electron double resonance
DRY: D^3.49^R^3.50^Y^3.51^
ECL: extracellular loop
EXSY: exchange spectroscopy
ICL: intracellular loop
fs: femtoseconds
GEF: guanine nucleotide exchange factor
GaMD: Gaussian-accelerated MD
GDP: guanosine diphosphate
GTP: guanosine triphosphate
GPCR: G protein-coupled receptor
GPCRdb: GPCR database
GPCRK or GRKs: GPCR kinase
GPMV: giant plasma membrane vesicle
H8: intracellular helix 8
HDX-MS: hydrogen–deuterium exchange mass spectrometry
HMA: hexamethylene amiloride
5-HT_1B_R: serotonin 1B receptor
HRF-MS: hydroxyl radical footprinting mass spectrometry
κOR: κ-opioid receptor
LCP: lipidic cubic phase
LiP-MS: limited proteolysis coupled to mass spectrometry
MS: mass spectrometry
MALDI-MS: high-throughput matrix-assisted laser desorption/ionization mass spectrometry
LRET: luminescence resonance energy transfer
M3R: muscarinic acetylcholine receptor M3
mB: monobromobimane
MC: Monte Carlo
μOR: μ opioid receptor
ms: milliseconds
NAM: negative allosteric modulator
nMS: native MS
NPxxY: N^7.49^P^7.50^xxY^7.53^
NTS1R: neurotensin receptor 1
NMR: nuclear magnetic resonance
OBS: orthosteric binding site
PAM: positive allosteric modulator
PET: photoinduced electron transfer
PIF: P^5.50^I^3.40^F^6.44^
PIP2 or PI­(4,5)­P2: phosphatidylinositol-4,5-bisphosphate
POPC: 1-palmitoyl-2-oleoyl-*sn*-glycero-3-phosphocholine
POPE: 1-palmitoyl-2-oleoyl-*sn*-glycero-3-phosphoethanolamine
POPG: 1-palmitoyl-2-oleoyl-*sn*-glycero-3-phosphoglycerol
POPS: 1-palmitoyl-2-oleoyl-*sn*-glycero-3-phosphoserine
PRE: paramagnetic relaxation enhancement
RHD: Ras-like domain
rHDLs: reconstituted high-density lipoproteins
Rho: rhodopsin
RMSD: root-mean-square deviation
SMF: single-molecule fluorescence
smFRET: single-molecule fluorescence resonance energy transfer
SPASM: Systematic Protein Affinity Strength Modulation
STD: saturation transfer difference
TIRF: total internal reflection fluorescence
TM: transmembrane
7TM: seven transmembrane α-helix
TMSF: 4-(trimethylsilyl)­phenylalanine
V2R: vasopressin type 2 receptor
YY: Y^5.58^---W^6.48^---Y^7.53^ or Y^5.58^---water---Y^7.53^
